# Asymmetric Total Synthesis of (−)‐Glycybridin B, a Pharmacophore Screened Candidate for Tubulin Binding

**DOI:** 10.1002/chem.202502228

**Published:** 2025-08-06

**Authors:** Alice Maiocchi, Maxim Shevelev, Zlata Boiarska, Juan Estévez‐Gallego, Francesca Bonato, Paolo Orlando, Alessandra Chinosi, Emanuele Marcone, Andrea Citarella, Dragos Horvath, Michel O. Steinmetz, Andrea E. Prota, Alexandre Varnek, Valerio Fasano, Daniele Passarella

**Affiliations:** ^1^ Department of Chemistry University of Milan ‐ Milan 20133 Italy; ^2^ Laboratory of Chemoinformatics University of Strasbourg Strasbourg 67081 France; ^3^ PSI Center for Life Sciences Villigen PSI 5232 Switzerland; ^4^ University of Basel Biozentrum Basel 4056 Switzerland

**Keywords:** glycybridin B, natural products, total synthesis, tubulin, virtual screening

## Abstract

Microtubule‐targeting agents (MTAs) represent a pivotal class of therapeutic compounds designed to disrupt microtubule dynamics, leading to cell cycle arrest and apoptosis in malignant cells. Nevertheless, their off‐target effects on healthy, rapidly dividing cells result in significant neurotoxicity and myelosuppression. Ongoing research aims to enhance their specificity and identify novel active scaffolds to minimize adverse effects and fight drug resistance. Searching for new potential tubulin binders with a pharmacophore screening method, we identified glycybridin B as a promising natural product. Here, we report the first total synthesis of this compound via a five‐step route, involving a key chalcone intermediate, along with its biological evaluation.

## Graphical abstract

Discovery and first total synthesis of (−)‐glycybridin B via a five‐step route utilizing a key chalcone intermediate. Initially identified through a pharmacophore screening as a potential tubulin binder, this natural product serves as a tool compound to explore structure‐activity relationships related to microtubule targeting and anticancer potential.

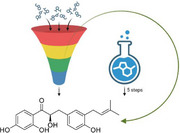

## 1 Introduction

Microtubule‐targeting agents (MTAs) are therapeutic compounds that interfere with the dynamics of microtubules as structural components of the cytoskeleton in eukaryotic cells. Microtubules play a critical role in various cellular processes, including maintenance of cell shape, intracellular transport, and, most importantly, the orchestration of cell division.^[^ ^1^ ^]^ Their physiological functions are highly regulated, and any disruption can lead to cell cycle arrest and apoptosis, making MTAs particularly relevant in cancer treatment.^[^ ^2^ ^]^ However, they can also lead to side effects due to their impact on normal, rapidly dividing cells, which can result in neurotoxicity, myelosuppression, and gastrointestinal issues.^[^ ^3^ ^]^ Research efforts focus on understanding the mechanisms of action of MTAs^[^ ^4^ ^]^ and on improving their specificity and efficacy to enhance their therapeutic potential while minimizing adverse effects and overcoming resistance mechanisms.

**Scheme 1:**
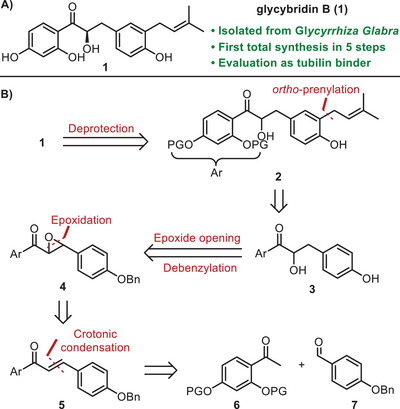
Retrosynthetic scheme of glycybridin B with the key reactions highlighted.

In this context, a challenging approach is the identification and optimization of novel or existing MTAs. While searching for new potential tubulin binders, the natural product glycybridin B (**1**) was identified as a promising molecule through pharmacophore screening, predicted to bind at the maytansine site of tubulin. Glycybridin B was first isolated in 2017 by Li and coworkers^[^ ^5^ ^]^ from the herbal medicine *Glycyrrhiza Glabra* as one of its bioactive compounds (Scheme 1A). Indeed, the clinical therapeutic benefits of liquorice are widely recognized due to its hepatoprotective, anti‐inflammatory, neuroprotective, antioxidant, and antiviral properties.^[^ ^6^, ^7^ ^]^ Given its notable properties, particularly its potential cytotoxic activity against cancer cells, and the absence of a reported synthesis in the literature, the total synthesis of glycybridin B was pursued.

## 2 Results and Discussion

### 2.1 Virtual Screening

Using the crystal structure of the tubulin–disorazole Z complex (PDB ID: 6FJM), we developed a structure‐based pharmacophore model of the maytansine binding site with LigandScout software.^[^ ^8^ ^]^ This model, incorporating key hydrophobic and hydrogen‐bonding interactions, was validated through structural alignment with an independent maytansine‐bound tubulin complex (PDB ID: 4TV8). Subsequently, the ChEMBL database (v26)^[^ ^9^ ^]^ was screened against this model, resulting in 1035 virtual hits. These candidates were docked into the maytansine site using PLANTS software,^[^ ^10^ ^]^ and 104 compounds were found to exhibit better docking scores than the reference ligand, disorazole. Cross‐referencing with ChEMBL bioassay data revealed six cytotoxic compounds acting by an unidentified mechanism. This set of molecules includes ChEMBL 562 005, which shows cytotoxicity against human HBI10A cells (Hepatitis C Virus infected cells); ChEMBL 3 764 095, a cytotoxic agent against human Bel7404 cells (human hepatocellular carcinoma); ChEMBL 3 088 172, which shows cytotoxicity against human A549 cells (human lung carcinoma); ChEMBL 1 589 498, which results from a Fluorescence Polarization counter screen qHTS toward Inhibitors of Tau Fibril Formation (this assay is used as a model for amyotrophic lateral sclerosis); ChEMBL 2 022 147, a cytotoxic agent against human HG23 cells (human Norwalk virus infected cells), and finally ChEMBL 4 076 273 (Glycybridin B), an antiproliferative agent against human MCF7 cells (human breast cancer). Among the six identified hits, glycibridin B (see an image of a minimized tubulin‐glycibriding complex in Supporting Information) demonstrated promising properties, including a docking score superior to that of disorazole and alignment with three pharmacophore features. Literature reports also support its antiproliferative activity against MCF7 breast cancer cells. Before progressing to synthesis, we investigated the structure–activity relationship of Glycybridin B through targeted in silico modifications. The discussed modifications concerned its core scaffold, as well as the substituents on the aromatic rings, focusing on a few main variations: a hydrogen or an alkyl chain instead of the prenyl moiety, a diol or an alkyl chain replacing the α‐hydroxyl ketone, and the R or S configuration of its chiral α‐hydroxyl group. Computational analysis of these modified molecules could not identify any improved docking score at the maytansine site with respect to parent glycybridin B; in fact, its prenyl substituent was identified as a crucial factor for binding interactions, as evidenced by the inability of its reduced form (isopentyl) and hydrogen‐containing variants to bind at this site; moreover, three out of five putative binding molecules contained a hydroxyketone scaffold, which may indicate its importance for binding, while the natural (*R*) configuration leads to a better binding score than the (*S*) one. In consideration of these results, a retrosynthetic pathway for glycybridin B was devised (Scheme 1B).

### 2.2 Chemistry

The retrosynthetic approach employed for the synthesis of glycybridin B is illustrated in Scheme 1B. Glycybridin B **1** should be obtained from its protected analogue **2**, derived from an *ortho*‐prenylation of intermediate **3**. The latter can be obtained through the opening of epoxide **4** and the simultaneous removal of the benzyl protecting group. Epoxide **4** should come from the epoxidation of chalcone **5**, which, in turn, should result from an aldolic condensation between protected ketone **6** and 4‐(benzyloxy)benzaldehyde **7**. To support the experimental synthesis efforts, we computationally evaluated 14 analogues of glycybridin B–seven intermediates already accessible through the proposed synthetic route and seven derivatives that could be readily obtained from them. Using PLANTS docking software and the previously defined maytansine binding site from the disorazole Z‐bound tubulin structure (PDB ID 6FJM), all 14 analogues were docked and assessed using the same scoring parameters applied to the original virtual screen. Interestingly, each analogue yielded a better docking score than the native ligand (disorazole Z), making it difficult to rank them solely on this basis. To further distinguish between these candidates, we employed blind docking, which assesses a ligand's ability to bind elsewhere on the protein, thereby revealing whether the maytansine site is indeed the ligand's preferred binding pocket. This approach identified two methoxy‐protected versions of glycybridin B, which consistently favored the maytansine site over other known binding sites and had been considered synthetically due to the more straightforward deprotection steps compared to the parent compound. Subsequent pharmacophore analysis confirmed that these ligands overlapped with five out of the ten features of the disorazole‐based pharmacophore model, suggesting that they maintain the essential interactions required for biological activity at the target site. These docking results therefore provided computational justification for the synthesis of this methoxy‐protected analogue, as it preserves a pharmacophore‐consistent binding mode while offering greater synthetic accessibility. However, to reduce problems in the final phenol deprotection, alternative efforts were centered on the methoxymethyl (MOM) group, possibly showing simpler removal protocols than the former and basic stability. The initial plan was to obtain glycybridin B in its racemic form to then switch to an enantioselective synthesis, thus providing the (R)‐configuration embedded in the natural product.

#### 2.2.1 Synthesis of Dimethoxy GLYCYBRIDIN B

**Scheme 2:**
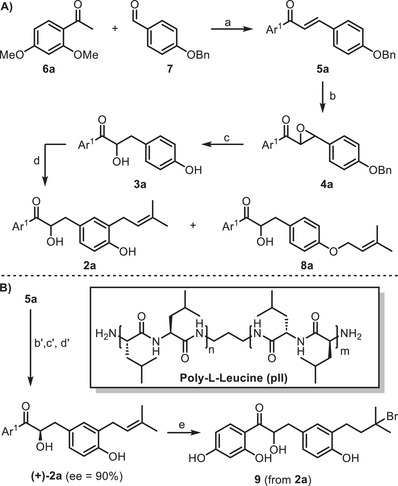
A) Synthesis of dimethoxy glycybridin B **2a**. a) 2 M NaOH, EtOH/H_2_O/THF, 40 °C, 28 hours, 88% yield; b) 30% H_2_O_2_, 2 M NaOH, MeOH, rt, 24 hours, 83% yield; c) H_2_, Pd‐BaSO_4_, AcOEt, rt, 28 hours, 83% yield; d) 3,3‐dimethylallyl bromide, 1.5 M KOH, H_2_O, 30 °C, 29 hours, 12% yield. e) BBr_3_, CH_2_Cl_2_, 0 °C to rt. B) Synthesis of enantioenriched dimethoxy glycybridin B **(+)‐2a**. b’) 30% H_2_O_2_, 5 M NaOH, poly‐L‐leucine, TBAB, toluene, 25 °C, 24 hours, 65% yield; c’) H_2_, Pd‐BaSO_4_, EtOAc, rt, 24 hours, 82% yield; d’) 3,3‐dimethylallyl bromide, 1.5 M KOH, H_2_O, 30 °C, 29 hours, 12% yield. e) BBr_3_, CH_2_Cl_2_, 0 °C to rt.

The first synthetic step involved the crotonic condensation between methoxy‐protected **6a** and **7** and was carried out under basic conditions (Scheme 2A).

Chalcone **5a** was obtained with an excellent yield after filtering the mixture. The following step entailed the formation of epoxide **4a** (step b), using H_2_O_2_ as the oxidizing agent under basic conditions; the desired epoxide **4a** was successfully obtained in very good yield. Subsequently, epoxide opening in β‐position was carried out through nucleophilic addition of molecular hydrogen. Epoxide **4a** was treated under hydrogen atmosphere using Pd‐BaSO_4_ as the catalyst, carefully optimizing reaction times to obtain benzyl group removal after epoxide opening while minimizing the undesired carbonyl reduction. The predicted β‐opening of the epoxide, resulting in α‐hydroxyketone **3a**, is in accordance with the benzylic nature of the cleaved bond. Intermediate **3a** possesses the skeleton of glycybridin B. The following step involved introducing the prenyl group using 3,3‐dimethylallyl bromide under basic conditions. *O*‐methoxy‐protected glycybridin B **2a** was obtained after the purification of the crude, along with *O*‐prenylated byproduct **8a** (similar results were already reported in the literature).^[^ ^11^ ^]^ Taking advantage of the synthetic steps developed for racemic dimethoxy glycybridin B **2a**, an α‐hydroxy carbonyl with (R)‐configuration could be sequentially generated from (2R,3S)‐epoxide **(−)‐4a**. When the epoxide is opened under molecular hydrogen, the configuration of its stereocenter should be retained.^[^ ^12^ ^]^ After extensive literature research,^[^ ^13^, ^14^ ^]^ Julia‐Colonna epoxidation was selected for the enantioselective epoxidation of chalcone **5a**, using poly‐L‐leucine (**pII**) as a chiral catalyst.^[^ ^15^ ^]^ Using reported literature conditions,^[^ ^14^ ^]^ chalcone **5a** was thus treated with H_2_O_2_ in 5 M NaOH, using **pII** as a chiral catalyst and tetrabutylammonium bromide (TBAB) as a phase transfer catalyst in the biphasic system (Scheme 2B). Epoxide **(−)‐4a** was successfully obtained with a good yield and 95% of enantiomeric excess (ee). Subsequently, the regioselective opening of epoxide **(−)‐4a** followed by prenylation resulted in the desired dimethoxy glycybridin B **(+)‐2a**. With this compound in hand, deprotection of the methoxy groups was attempted to obtain glycybridin B. Unfortunately, initial attempts on **2a** with aluminium trichloride (AlCl_3_) or sodium ethanethiolate (EtSNa) were unsuccessful, whereas the use of boron tribromide (2 or 4 equivalents) led mainly to brominated product **9**, the result of undesired hydrobromination of the double of glycybridin.

#### 2.2.2 Synthesis of Glycybridin B

**Scheme 3:**
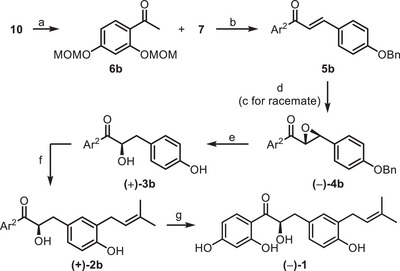
Synthesis of racemic and enantiopure glycybridin B: a) MOM‐Br, NaH, dry DMF, 0 °C to rt, 4 hours, 65% yield; b) NaOH, EtOH/H_2_O/THF, 40 °C, 48 hours, 76% yield; c) 30% H_2_O_2_, 2 M NaOH, MeOH, rt, 26 hours, 69% yield; d) 30% H_2_O_2_, 5 M NaOH, TBAB, poly‐L‐leucine, toluene, 25 °C, 24 hours, 68% yield; e) H_2_, Pd‐BaSO_4_, EtOAc, rt, 20 hours, (+)**3b**: 74% yield, (±)**3b**: 66% yield; f) 3,3‐dimethylallyl bromide, 1.5 M KOH, H_2_O, 30 °C, 24 hours, (+)**2b**: 14% yield, **2b**: 14% yield; g) DOWEX, MeOH, rt, 48 hours, (‐)**1**: 23% yield, **1**: 20% yield.

As an alternative strategy, the methoxymethyl group was considered to address the challenges encountered in the removal of methoxy groups. Given that the entire synthetic pathway takes place predominantly under basic conditions, acid‐removable protecting groups were deemed appropriate. The synthesis started by protecting commercially available 1‐(2,4‐dihydroxyphenyl)ethan‐1‐one **10** with MOM‐Br under basic conditions in good yield (step a, Scheme 3). The resulting protected ketone **6b** was reacted with 4‐(benzyloxy)benzaldehyde **7** in a crotonic condensation to form chalcone **5b**, obtained in a lower yield compared to **5a**, probably due to the increased steric hindrance provided by the MOM groups.

Then, **5b** underwent epoxidation in racemic or asymmetric fashion, obtaining racemic **4b** (suitable for HPLC analysis) or enantioenriched **(−)‐4b**, respectively. In particular, racemic epoxide **4b** was obtained in good yield by reacting chalcone **5b** with 30% aqueous H_2_O_2_ in the presence of NaOH (step c). Conversely, the enantioselective synthesis was performed with the same reagents but using TBAB as the phase transfer catalyst and poly‐L‐leucine **pII** as the chiral catalyst (step d). The desired enantiopure epoxide **(−)‐4b** was obtained with a good yield and with 89% *ee*. Then, regioselective opening of both epoxides yielded α‐hydroxyketone **3b** or its enantioenriched **(+)‐3b** (with the desired (*R*)‐configuration), respectively. Subsequently, prenylation of **3b** or **(+)‐3b** furnished MOM‐protected glycybridin B **2b** or **(+)‐2b**, together with the *O*‐prenylated byproduct. Finally, removal of MOM groups from protected **(+)‐2b** and **2b** led to the target glycybridin B **(−)‐1** and **1**, respectively. Notably, MOM‐protected glycybridin B **(+)‐2b** was first treated with 4 M HCl in dioxane, but such a strong acidic condition favored intramolecular *O*‐cyclization with the prenyl moiety. The weaker CSA was subsequently employed; once again, only the cyclized product was isolated, together with residual starting material, demonstrating that the intramolecular cyclization in acidic protic conditions occurs very fast. Subsequently, it was decided to move from a protic acid to an acid resin; in this case, the reaction led to the disappearance of starting material with the concomitant formation of the mono‐deprotected and fully deprotected products. Both were isolated through flash chromatography, successfully obtaining glycybridin B **(−)‐1**. The same strategy was applied for the synthesis of racemic analogue **1**.

### 2.3 Biological Evaluation

**Figure 1:**
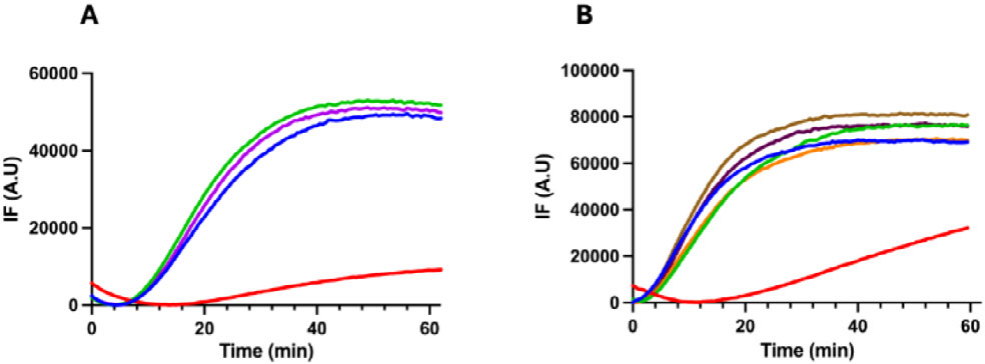
Effects of glycybridines on tubulin polymerization. Time course fluorimetry of tubulin polymerization in the presence of: A. 30 µM of enantioenriched glycybridine B **(−)‐1** (green line) and its racemic mixture **1** (purple line). B. 40 µM of glycybridine B derivatives **3a** (green line), **2a** (orange line), **(+)‐3a** (brown line), and **(+)2‐a** (dark purple line). The effects on tubulin polymerization are compared with control vehicle (1% DMSO, blue line) and 40 µM Nocodazole (red line). Results correspond to the average result obtained from three independent experimental replicates.

Microtubule destabilizing agents preclude tubulin assembly by blocking the addition of new tubulin subunits onto the microtubule. To assess the inhibitory effect of the newly synthetized glycybridine‐derivatives on tubulin polymerization (Figure 1), we monitored the inhibition of tubulin assembly by fluorimetry, as previously described.^[^ ^16^, ^17^, ^18^ ^]^ Apparently, none of the assayed compounds displayed a significant effect on tubulin polymerization dynamics under the assayed conditions.

## 3 Conclusions

In conclusion, the first total synthesis of glycybridin B has been achieved. The synthetic strategies developed, including both racemic and enantioselective routes utilizing various protecting groups, demonstrate the feasibility of producing this natural product with a 5‐step route under user‐ and environment‐friendly conditions. Despite the successful synthesis and structural validation, biological evaluations revealed that the key intermediates and glycybridin B itself do not significantly inhibit tubulin polymerization under the tested conditions. These findings underscore the complexity of translating in silico predictions into biological activity and suggest that further modifications may be necessary to fully elucidate the therapeutic potential of glycybridin B derivatives. Moving forward, optimizing these compounds for enhanced binding affinity and biological efficacy remains a critical step toward developing novel MTAs agents with improved selectivity and reduced side effects.

## 4 Experimental Section

### 4.1 Virtual Screening

To employ the pharmacophore‐based virtual screening strategy, a search of the Protein Data Bank (PDB) identified six high‐resolution structures of tubulin complexed with ligands bound at the maytansine site (PDB IDs: 6FJM, 6FII, 6FJF, 4TV8, 4TUY, 4TV9). Re‐docking experiments using PLANTS software^[^ ^10^ ^]^ were conducted to assess the reproducibility of docking poses for each cocrystallized ligand. The protein and ligand files were prepared using the SPORES software to ensure proper protonation states, tautomeric forms, and 3D geometry optimization prior to docking. Ligands were stripped from their native complexes, and the resulting protein structures were cleaned of water, ions, and minor molecules. Binding site definition encompassed all protein atoms within 12 Å of the ligand's center of mass. The re‐docking accuracy was measured via the root mean square deviation of atomic position metric (RMSD); disorazole demonstrated the most favorable performance (3.05 Å), despite minor deviations due to its flexible macrocyclic region. Therefore, the 6FJM‐disorazole complex was selected for pharmacophore model development using LigandScout,^[^ ^8^ ^]^ yielding a model comprising six hydrophobic features, three hydrogen bond acceptors, one hydrogen bond donor, and exclusion volumes matching the binding site contours.

To validate the pharmacophore model, structural alignment of the 4TV8 maytansine‐bound tubulin with the 6FJM‐disorazole complex was conducted in ChimeraX.^[^ ^19^ ^]^ The superposition revealed strong spatial concordance of key features, particularly those interacting with βASN101 and βVAL181 residues. This confirmed the robustness of the pharmacophore model and justified its use for virtual screening. Subsequently, the ChEMBL database (v26, 1 771 509 molecules)^[^ ^9^ ^]^ was prepared for screening. All compounds were standardized using ChemAxon's Standardizer following a multistep process including dearomatization, salt and mixture removal, neutralization, and tautomer generation. Molecular conformers were then generated using LigandScout's iCon tool, with up to 200 diverse conformations per compound, enabling comprehensive pharmacophore mapping.

Pharmacophore screening was executed in LigandScout using a “pharmacophore fit” scoring method, matching a minimum of three features per compound. To optimize efficiency, the “stop after first matching conformation” and exclusion volume clash checks were enabled. This screening yielded 1035 virtual hits with fit scores exceeding 64. These hits were further evaluated by docking into the maytansine site of tubulin (from 6FJM) using PLANTS. Docking utilized the chemplp scoring function and generated ten poses per compound, selecting the best‐scoring pose for comparison. Of the 1035 candidates, 104 exhibited docking scores superior to disorazole and aligned with at least three pharmacophore features.

To explore the structure‐activity relationship of ChEMBL4076273 by a focused optimization study, six scaffold variants and 1444 modifications of the substituents were generated using RDKit, examining changes to the central linker (length, functionality, and stereochemistry) and peripheral aromatic substituents (hydroxyl group positioning and replacements with fluoro‐, chloro‐, methoxy‐, amino‐, and nitro‐ functional groups). All variants were docked into the maytansine site and adjacent pockets using PLANTS, and the best‐scoring poses were analyzed for binding potential.

To support the design and synthesis of Glycybridin B analogues, a set of 14 molecules was selected for computational evaluation. This set included seven synthetic intermediates already accessible within the proposed retrosynthetic route and seven structurally related derivatives considered synthetically feasible. All compounds were subjected to docking using PLANTS software, following the same docking protocol applied in the primary virtual screening campaign. The docking simulations were conducted against the maytansine binding site of tubulin, defined as all atoms within a 12 Å radius from the center of mass of disorazole in the 6FJM PDB structure. Each ligand was docked with ten generated poses, and the pose with the lowest docking score was retained for subsequent analysis.

Since all 14 analogues exhibited better docking scores than disorazole, we additionally performed blind docking to assess the binding site preference of each molecule. In this approach, the same α,β‐tubulin heterodimer (chains C and D from PDB ID: 6FJM) was used, with all solvent molecules and heteroatoms removed. The docking grid was expanded to encompass the entire protein, defined as all atoms within 60 Å of the geometric center formed by four randomly selected residues on the interdimer surface. Blind docking simulations were also carried out using PLANTS, with an increased sampling depth of 40 docking poses per ligand to capture a wider range of potential binding sites.

The docking results were analyzed to determine whether each compound preferentially docked to the maytansine site or to alternative pockets. Two methoxy‐protected ligands were found to favor the maytansine binding site across the majority of their poses. These two compounds were further analyzed by comparing their top docking poses to the previously derived disorazole‐based pharmacophore model using LigandScout. Pharmacophore feature mapping revealed that both ligands matched five of the ten pharmacophoric features, indicating potential for functional activity at the target site.

### 4.2 Synthesis

Oven‐dried glassware was used to carry out chemical reactions, and dry solvents under a nitrogen atmosphere were employed. Solvents were purchased from Sigma Aldrich and used as such. Chemical reagents were purchased from Sigma Aldrich, Fluorochem, and TCI and used without further purification. Reaction monitoring by thin layer chromatography (TLC) entailed Merck precoated 60F254 plates, using UV light at 254 nm as a direct detection method, or by staining with a phosphomolybdic acid ethanolic solution. The purification of intermediates and final products was carried out by flash chromatography using high‐purity‐grade silica gel (Merck Grade, pore size 60 Å, 230–400 mesh particle size, Sigma‐Aldrich, Milan, Italy) as a stationary phase. Alternatively, purification was performed by a Biotage system using Biotage Sfar Silica D cartridges (10 or 25 g) for direct phase chromatography. ^1^H‐NMR and ^13^C‐NMR spectra were recorded in CDCl_3_ on a Bruker AC 400 instrument. Chemical shifts (δ) for proton and carbon signals were referenced to the solvent (CDCl_3_: 1H‐NMR: 7.26 ppm, ^13^C‐NMR: 77.16 ppm; CD_3_CN: ^1^H‐NMR: 1.94 ppm, ^13^C‐NMR: 1.32 ppm; CD_3_OD: ^1^H‐NMR: 3.31 ppm; dmso‐d^[^ ^6^ ^]^: ^1^H‐NMR: 2.50 ppm) and expressed in parts per million (ppm). HRMS spectra were recorded using electrospray ionization (ESI) technique on an FT‐ICR APEXII (Bruker Daltonics, Bremen, Germany). Analytical HPLC was performed on an Agilent 1100 Series System.

*Synthesis of (E)‐3‐(4‐(benzyloxy)phenyl)‐1‐(2,4‐dimethoxyphenyl)prop‐2‐en‐1‐one 5a*. A solution of 4‐benzyloxybenzaldehyde 7 (1.00 g, 5.55 mmol, 1.0 eq) and 1‐(2,4‐dimethoxyphenyl)ethan‐1‐one 6a (1.18 g, 5.55 mmol, 1.0 eq) in 6:4 EtOH:THF (24.2 mL) was added dropwise to a solution of NaOH (2.04 g, 51 mmol, 9.2 eq) in 6:4 EtOH:H_2_O (19.2 mL). The reaction was stirred at 40 °C for 24 hours, while monitoring by TLC (eluent mixture: 7:3 *n*‐Hex:EtOAc). After 24 hours, additional solid NaOH (0.22 g, 5.55 mmol, 1.0 eq) was added, and the mixture was stirred for an additional 4 hours. Then, it was cooled down to 0 °C observing the precipitation of a yellow solid. The solid was filtered, obtaining the pure product 5a^[^ ^20^ ^]^ (1.51 g, 4.03 mmol), while the mixture was extracted with CH_2_Cl_2_ (3 × 50 mL). The collected organic phases were dried over Na_2_SO_4_, filtered, and the solvent was removed under reduced pressure. The crude was purified with flash chromatography (silica gel, eluent mixture 8:2 *n*‐Hex:EtOAc) providing additional pure chalcone 5a (0.313 g, 0.837 mmol). The product was obtained as a yellow solid (88% overall yield). MS (ESI) *m/z* [M + H]^+^ = 375.1598 (calculated for C_24_H_23_O_4_, 375.1596). ^1^H NMR (400 MHz, CDCl_3_) δ 7.74 (d, *J *= 8.6 Hz, 1H), 7.64 (d, *J* = 15.7 Hz, 1H), 7.54 (d, *J* = 8.7 Hz, 2H), 7.47–7.30 (m, 6H), 6.99 (d, *J* = 8.7 Hz, 2H), 6.56 (dd, *J *= 8.6, 2.3 Hz, 1H), 6.50 (d, *J* = 2.3 Hz, 1H), 5.11 (s, 2H), 3.90 (s, 3H), 3.87 (s, 3H). ^13^C NMR (101 MHz, CDCl_3_) δ 190.7, 164.0, 160.4, 160.3, 142.0, 136.6, 132.8, 130.0, 128.7, 128.4, 128.2, 127.5, 125.2, 122.5, 115.2, 105.1, 98.7, 70.1, 55.8, 55.6.

*Synthesis of (3‐(4‐(benzyloxy)phenyl)oxiran‐2‐yl)(2,4‐dimethoxy‐phe‐nyl)methanone 4a*. 30% H_2_O_2_ (1.46 mL, 14.3 mmol, 1.2 eq) and 2 M aqueous NaOH (10.7 mL, 21.4 mmol, 1.8 eq) were added to a stirred solution of 5a (4.5 g, 12.0 mmol, 1.0 eq) in MeOH (201 mL) at rt. The reaction mixture was stirred at rt for 22 hours and monitored by TLC (eluent mixture: 6:4 *n*‐Hex:EtOAc). Then, additional quantities of 30% H_2_O_2_ (0.502 mL, 4.9 mmol, 0.4 eq) and 2 M aqueous NaOH (4.0 mL, 8.0 mmol, 0.7 eq) were added. After 24 hours, the solvent was concentrated under reduced pressure, and the crude was extracted with EtOAc (3 × 50 mL). The combined organic layers were washed with distilled H_2_O (50 mL), brine (50 mL) and dried over Na_2_SO_4_. The solvent was then concentrated under vacuum, and the crude was purified with Biotage (eluent: *n*‐Hex/EtOAc from 10% EtOAc to 80%) affording pure epoxide 4a (3.24 g, 8.21 mmol, 83% yield) as a white solid. MS (ESI) *m/z* [M + H]^+^ = 391.1561 (calculated for C_24_H_23_O_5_, 391.1546). ^1^H NMR (400 MHz, CDCl_3_) δ 7.89 (d, *J* = 8.7 Hz, 1H), 7.48–7.32 (m, 5H), 7.30 (d, *J* = 8.7 Hz, 2H), 6.99 (d, *J* = 8.7 Hz, 2H), 6.57 (dd, *J* = 8.7, 2.2 Hz, 1H), 6.40 (d, *J* = 2.2 Hz, 1H), 5.09 (s, 2H), 4.31 (d, *J* = 2.0 Hz, 1H), 3.92 (d, *J* = 2.0 Hz, 1H), 3.86 (s, 3H), 3.57 (s, 3H). ^13^C NMR (101 MHz, CDCl_3_) δ 193.7, 165.6, 161.7, 159.3, 136.9, 132.9, 129.1, 128.7, 128.2, 127.6, 127.3, 119.4, 114.7, 105.9, 98.3, 70.2, 64.6, 59.5, 55.8, 55.7.

*Synthesis of 1‐(2,4‐dimethoxyphenyl)‐2‐hydroxy‐3‐(4‐hydroxyphenyl) propan‐1‐one 3a*. 5% Pd‐BaSO_4_ (300 mg, 0.14 mmol, 0.4 eq) was added to a solution of 4a (144 mg, 0.369 mmol, 1.0 eq) in EtOAc (55 mL). The reaction was stirred at rt under hydrogen atmosphere and monitored by TLC (eluent mixture: 5:5 *n*‐Hex:EtOAc). Upon completion, the mixture was filtered on a Celite pad, and the solvent was removed under reduced pressure. The crude product was purified by Biotage (eluent mixture *n*‐Hex:EtOAc from 12% EtOAc to 100%) providing pure phenol 3a (92 mg, 0.384 mmol, 83% yield) as a white solid. MS (ESI) *m/z* [M + H]^+^ = 303.1255 (calculated for C_17_H_19_O_5_, 303.1233). ^1^H NMR (400 MHz, CD_3_CN) δ 7.79 (dd, *J* = 8.4, 0.8 Hz, 1H), 6.99 (d, *J* = 8.5 Hz, 2H), 6.72 (s, 1H), 6.70–6.66 (m, 2H), 6.63 (d, *J* = 8.4 Hz, 2H), 5.18 (dq, *J* = 8.2, 3.6 Hz, 1H), 3.95 (s, 3H), 3.88 (s, 3H), 3.82–3.67 (m, 1H), 2.99 (dd, *J* = 14.0, 3.6 Hz, 1H), 2.52 (dd, *J* = 14.0, 7.8 Hz, 1H). ^13^C NMR (101 MHz, CD_3_CN) δ 201.5, 166.6, 162.2, 156.3, 134.0, 131.5, 130.5, 118.3, 115.7, 107.5, 99.3, 78.2, 56.6, 56.5, 40.8.

*Synthesis of 1‐(2,4‐dimethoxyphenyl)‐2‐hydroxy‐3‐(4‐hydroxy‐3‐(3‐me‐thylbut‐2‐en‐1‐yl)phenyl) propan‐1‐one 2a*. 3a (189 mg, 0.630 mmol, 1.0 eq) was dissolved in 1.5 M aqueous KOH solution (0.42 mL, 0.630 mmol, 1.0 eq) at rt. 3,3‐Dimethyl allyl bromide (102 µL, 0.879 mmol, 1.4 eq) and additional 1.5 M KOH (0.42 mL, 0.630 mmol, 1.0 eq) were added in parallel, in two portions over 1 hour under vigorous stirring at 30 °C. The reaction was monitored by TLC (eluent mixture: 5:5 *n*‐Hex:EtOAc, developed in potassium permanganate). After 4 hours, an additional 1.5 M aqueous KOH (0.21 mL, 0.315 mmol, 0.5 eq) was added. After 27 hours, the reaction was acidified with 1 M HCl and extracted with EtOAc (3 × 5 mL). The organic layer was dried over anhydrous Na_2_SO_4_ and concentrated under reduced pressure. The crude was purified by flash chromatography (silica gel, eluent mixture 7:3 *n*‐Hex:EtOAc) providing pure prenyl phenol 2a (26 mg, 0.691 mmol, 12% yield) as a yellow oil. MS (ESI) *m/z* [M + H]^+^ = 371.1865 (calculated for C_22_H_27_O_5_, 371.1859). ^1^H NMR (400 MHz, CDCl_3_) δ 7.93 (d, *J* = 8.8 Hz, 1H), 6.96–6.88 (m, 2H), 6.70 (d, *J* = 7.9 Hz, 1H), 6.63 (dd, *J* = 8.8, 2.2 Hz, 1H), 6.52 (d, *J* = 2.2 Hz, 1H), 5.33 (dq, *J* = 6.9, 3.7 Hz, 2H), 3.96 (s, 3H), 3.92 (s, 3H), 3.32 (d, *J* = 7.3 Hz, 2H), 3.07 (dd, *J* = 14.1, 3.7 Hz, 1H), 2.64 (dd, *J* = 14.1, 7.3 Hz, 1H), 1.79 (s, 3H), 1.79 (s, 3H). ^13^C NMR (101 MHz, CDCl_3_) δ 200.3, 165.6, 161.0, 152.9, 134.2, 133.9, 130.8, 129.7, 128.3, 126.6, 122.1, 117.3, 115.4, 106.0, 98.3, 77.3, 55.7, 55.6, 40.3, 29.6, 25.8, 17.9.

*Synthesis of poly‐L‐leucine*. (*S*)‐4‐isobutyloxazolidine‐2,5‐dione (1 g, 6.36 mmol, 1.0 eq) was dissolved in anhydrous toluene (15 mL) at rt. The initiator 1,3‐diaminopropane (5.9 µL, 0.08 mmol, 0.0125 eq) was then added under stirring. Subsequently, the reaction mixture was gradually heated to 80 °C over 1 hour. During this period CO_2_ was liberated. Stirring was maintained for 2 hours at 80 °C. After cooling down to 60 °C, MeOH (10 mL) was added, and the resulting heterogeneous mixture was stirred for 2 hours at 60 °C before the solid was filtered off. Drying furnished pure poly‐L‐leucine (507 mg) as a white solid.

*Synthesis of ((2R,3S)‐3‐(4‐(benzyloxy)phenyl)oxiran‐2‐yl)(2,4‐dimetho‐xyphenyl)methanone (−)‐4a*. A flask was successively charged with TBAB (52 mg, 0.160 mmol, 0.2 eq), poly‐L‐leucine (22 mg), toluene (4.0 mL) and 5 M aqueous NaOH (0.418 mL, 2.09 mmol, 2.6 eq). After cooling to 15 °C, 30% H_2_O_2_ (0.816 mL, 8.04 mmol, 10.0 eq) was added, and the resulting heterogeneous mixture was warmed to 25 °C for 1 hour. Then, chalcone 5a (300 mg, 0.804 mmol, 1.0 eq) was added as a solid. Subsequently, the reaction was stirred for 23 hours in the dark at 25 °C, monitored by TLC (eluent mixture: 6:4 *n*‐Hex:EtOAc). Upon completion, the reaction mixture was diluted with EtOAc (10 mL) and quenched by the addition of ice‐cold 20% aqueous NaHSO_3_ solution (6 mL). After the addition of H_2_O (2 mL), phase separation furnished an organic layer containing the target epoxide and an aqueous layer containing insoluble poly‐L‐leucine. The aqueous phase was extracted with EtOAc (2 × 5 mL), the collected organic layers were dried over Na_2_SO_4_, filtered, and concentrated under vacuum. The obtained crude was purified by flash chromatography (silica gel, eluent mixture 7:3 *n*‐Hex:EtOAc), achieving enantiopure epoxide *(−)‐*4a (203 mg, 0.519 mmol, 65% yield, ee% = 95%) as a white solid. HPLC (Chiralpak AD, *n*‐Hex/i‐PrOH from 20:80 to 0:100, flow rate 0.8 mL/min): tr(2*S*,3*R*) = 15.8 minutes, tr(2*R*,3*S*) = 18.8 minutes. [α]^30^_D_ = ‐32.9 (c 0.41, CHCl_3_).

*Synthesis of (R)‐1‐(2,4‐dimethoxyphenyl)‐2‐hydroxy‐3‐(4‐hydroxy‐phenyl)propan‐1‐one (+)‐3a*. The same procedure as for 3a was used, starting from *(−)‐*4a (693 mg, 1.77 mmol) and obtaining (+)‐3a in 82% yield (440 mg, 1.46 mmol) as a white solid. All NMR and MS data are in agreement with the ones of the racemic compound. [α]^30^_D_ = + 56.6 (c 0.50, CHCl_3_).

*Synthesis of (R)‐1‐(2,4‐dimethoxyphenyl)‐2‐hydroxy‐3‐(4‐hydroxy‐3‐(3‐methylbut‐2‐en‐1‐yl)phe‐nyl)propan‐1‐one (+)‐2a*. The same procedure as for 2a was used, starting from (+)‐3a (205 mg, 0.683 mmol) and obtaining (+)‐2a in 12% yield (28 mg, 0.074 mmol) as a yellow oil. All NMR and MS data are in agreement with the ones of the racemic compound. [α]^30^_D_ = + 36.3 (c 0.44, CHCl_3_).

*Synthesis of 1‐(2,4‐bis(methoxymethoxy)phenyl)ethan‐1‐one 6b*. A solution of 2,4‐dihydroxyacetophenone (500 mg, 3.29 mmol, 1.0 eq) in dry DMF (10.2 mL) was added to a slurry of NaH (55% in mineral oil, 545 mg, 12.5 mmol, 3.8 eq) in dry DMF (8.19 mL) at 0 °C over a period of 20 minutes under N_2_ atmosphere, and the mixture was stirred for 1 hour at rt. Then, the reaction mixture was cooled down to 0 °C and MOM‐Br (0.803 mL, 9.86 mmol, 3.0 eq) was added slowly over 15 minutes. The mixture was then stirred for 4 hours at rt, monitored by TLC (eluent mixture: 7:3 *n*‐Hex:EtOAc). After the disappearance of the starting material, it was poured into ice‐cold H_2_O (10 mL) and extracted with EtOAc (3 × 15 mL). The combined organic layers were washed with distilled H_2_O (15 mL), brine (15 mL) and dried with anhydrous Na_2_SO_4_, filtered and concentrated under vacuum. The crude was purified with Biotage (eluent: *n*‐Hex/EtOAc from 7% EtOAc to 80%) affording pure bis‐MOM ketone 6b (530 mg, 2.14 mmol, 65% yield) as a colorless oil. ^1^H NMR (400 MHz, CDCl_3_) δ 7.81 (d, *J* = 8.7 Hz, 1H), 6.85 (d, *J* = 2.3 Hz, 1H), 6.75 (dd, *J* = 8.7, 2.3 Hz, 1H), 5.30 (s, 2H), 5.23 (s, 2H), 3.55 (s, 3H), 3.51 (s, 3H), 2.64 (s, 3H). Spectroscopic data are consistent with those reported in literature.^[^ ^21^ ^]^

*Synthesis of (E)‐3‐(4‐(benzyloxy)phenyl)‐1‐(2,4‐bis(methoxy‐methoxy)phenyl)prop‐2‐en‐1‐one 5b*. A solution of 4‐benzyloxybenzaldehyde 7 (1.44 g, 6.78 mmol, 1.4 eq) and ketone 6b (1.165 g, 4.84 mmol, 1.0 eq) in 6:4 EtOH:THF (30 mL) was added dropwise to a solution of NaOH (2.52 g, 63.0 mmol, 13.0 eq) in 6:4 EtOH:H2O (24 mL). The reaction was stirred at 40 °C for 24 hours, monitored by TLC (eluent mixture: 7:3 *n*‐Hex:EtOAc). After 24 hours, additional solid NaOH (0.775 g, 19.0 mmol, 4.0 eq) was added. After an additional 4 hours of stirring, the reaction mixture was cooled down to 0 °C observing the precipitation of a yellow solid. The solvent was evaporated, H_2_O (30 mL) and CH_2_Cl_2_ (30 mL) were added, the aqueous phase was extracted with CH_2_Cl_2_ (3 × 50 mL). The collected organic layers were dried over anhydrous Na_2_SO_4_, filtered, and concentrated under reduced pressure. The crude was purified with Biotage (eluent: *n*‐Hex/EtOAc from 6% EtOAc to 60%) affording pure chalcone 5b (1.60 g, 3.68 mmol, 76% yield) as a yellow solid. MS (ESI) *m/z* [M + H]^+^ = 435.1824 (calculated for C_26_H_27_O_6_, 435.1808). ^1^H NMR (400 MHz, CDCl_3_) δ 7.70–7.58 (m, 2H), 7.54 (d, *J* = 8.7 Hz, 2H), 7.47–7.29 (m, 6H), 6.99 (d, *J* = 8.7 Hz, 2H), 6.85 (d, *J* = 2.3 Hz, 1H), 6.77 (dd, *J* = 8.6, 2.3 Hz, 1H), 5.24 (s, 2H), 5.22 (s, 2H), 5.11 (s, 2H), 3.50 (s, 3H), 3.49 (s, 3H). ^13^C NMR (101 MHz, CDCl_3_) δ 191.4, 161.2, 160.6, 157.5, 142.7, 136.6, 132.2, 130.1, 128.8, 128.4, 128.3, 127.6, 125.2, 124.4, 115.4, 109.3, 103.8, 95.2, 94.4, 70.2, 56.7, 56.4.

*Synthesis of ((2R,3S)‐3‐(4‐(benzyloxy)phenyl)oxiran‐2‐yl‐2,4‐bis(methoxymethoxy)phenyl)methan‐one (−)‐4b*. A flask was successively charged with TBAB (237 mg, 3.69 mmol, 0.2 eq), poly‐L‐leucine (108 mg, 0.009 mmol, 0.003 eq), toluene (12.5 mL) and 5 M aqueous NaOH (3.85 mL, 19.2 mmol, 5.2 eq) and stirred. After cooling to 15 °C, 30% H_2_O_2_ (9.23 mL, 90.4 mmol, 24.5 eq) was added, and the resulting heterogeneous mixture was warmed to 25 °C for 1 hour before chalcone 5b (1.6 g, 3.69 mmol, 1.0 eq) dissolved in toluene (6.0 mL) was added. Then, the reaction was stirred for 24 hours in the dark at 25 °C monitoring by TLC (eluent mixture: 6:4 *n*‐Hex:EtOAc). Upon completion, the reaction mixture was diluted with EtOAc (10 mL) and quenched by the addition of ice‐cold 20% aqueous NaHSO_3_ (10 mL). After addition of H_2_O (20 mL), phase separation furnished an organic layer containing the target epoxide and an aqueous layer containing insoluble poly‐L‐leucine. The aqueous phase was extracted with EtOAc (2 × 20 mL) and the collected organic layers were dried over Na_2_SO_4_, filtered, and concentrated under vacuum. The obtained crude was purified by flash chromatography (silica gel, eluent mixture 7:3 *n*‐Hex:EtOAc) achieving enantiopure epoxide *(−)‐*4b (1.08 mg, 2.39 mmol, 68% yield, ee% = 89%) as a yellow solid. HPLC (Chiralpak AD, λ 220 nm, Hex/i‐PrOH from 20:80 to 0:100, flow rate 0.8 mL/min): tr(2*S*,3*R*) = 13.9 minutes, tr(2*R*,3*S*) = 16.7 minutes. [α]^30^_D_ = ‐104.3 (c 0.56, CHCl_3_). MS (ESI) *m/z* [M + H]^+^ = 451.1752 (calculated for C_26_H_27_O_7_, 451.1757). ^1^H NMR (400 MHz, CDCl_3_) δ 7.85 (dd, *J* = 8.6, 0.9 Hz, 1H), 7.47–7.31 (m, 5H), 7.28 (d, *J* = 8.5 Hz, 2H), 6.98 (dd, *J* = 8.5, 1.1 Hz, 2H), 6.79–6.74 (m, 2H), 5.20 (s, 1H), 5.10 (s, 2H), 4.89 (dd, *J* = 6.9, 0.9 Hz, 1H), 4.82 (dd, *J* = 6.9, 0.9 Hz, 1H), 4.30 (d, *J* = 2.0 Hz, 1H), 3.92 (d, *J* = 2.0 Hz, 1H), 3.47 (s, 2H), 3.10 (s, 2H). ^13^C NMR (101 MHz, CDCl_3_) δ 193.0, 162.9, 159.4, 159.2, 136.8, 132.5, 129.0, 128.8, 128.2, 127.6, 127.3120.6, 115.3, 109.6, 102.5, 94.6, 94.4, 70.2, 65.0, 59.6, 56.5, 56.5.

*Synthesis of (3‐(4‐(benzyloxy)phenyl)oxiran‐2‐yl)(2,4‐bis(methoxymethoxy)phenyl)methanone 4b*. 30% H_2_O_2_ (46 µL, 0.45 mmol, 3.6 eq) was added to a mixture of chalcone 5b (50 mg, 0.125 mmol, 1.0 eq) in MeOH (2.08 mL) and 2 M aqueous NaOH (337 µL, 0.675 mmol, 5.4 eq). The solution was stirred at rt monitoring by TLC (eluent: *n*‐Hex/EtOAc 5:5, developed in potassium permanganate). After 6 hours, the solvent was evaporated under vacuum and the residue was extracted with EtOAc (3 × 10 mL). The combined organic layers were washed with distilled H_2_O (10 mL), brine (10 mL) and dried with anhydrous Na_2_SO_4_, filtered, and concentrated under vacuum. The crude was purified by Biotage (eluent: *n*‐Hex/EtOAc from 12% EtOAc to 100%) affording pure epoxide 4b (39 mg, 0.086 mmol, 69% yield) as a yellow oil. HPLC (Chiralpak AD, λ 220 nm, *n*‐Hex/i‐PrOH from 20:80 to 0:100, flow rate 0.8 mL/min): tr(2*S*,3*R*) = 14.0 minutes, tr(2*R*,3*S*) = 17.0 minutes.

*Synthesis of (R)‐1‐(2,4‐bis(methoxymethoxy)phenyl)‐2‐hydroxy‐3‐(4‐hydroxyphenyl)propan‐1‐one (+)‐3b*. 5% Pd‐BaSO_4_ (912 mg, 0.43 mmol, 0.35 eq) was added to a solution of *(−)‐*4b (550 mg, 1.21 mmol, 1.0 eq) in EtOAc (174 mL). The reaction flask was stirred at rt under a hydrogen atmosphere, and the reaction was monitored by TLC (eluent mixture: 6:4 *n*‐Hex:EtOAc). Upon completion, the reaction mixture was filtered on a Celite pad and concentrated under reduced pressure. The crude product was purified by Biotage (eluent mixture *n*‐Hex:EtOAc from 12% EtOAc to 60%), providing pure α‐hydroxyketone (+)‐3b (323 mg, 0.892 mmol, 74% yield) as a white solid. [α]^30^_D_ = +56.6 (c 0.50, CHCl_3_). MS (ESI) *m/z* [M + H]^+^ = 363.1455 (calculated for C_19_H_23_O_7_, 363.1444). ^1^H NMR (400 MHz, CDCl_3_) δ 7.84 (d, *J* = 8.8 Hz, 1H), 6.98 (d, *J* = 8.5 Hz, 2H), 6.89 (d, *J* = 2.3 Hz, 1H), 6.79 (dd, *J* = 8.8, 2.3 Hz, 1H), 6.65 (d, *J* = 8.5 Hz, 2H), 5.38 (dd, *J* = 7.1, 3.9 Hz, 1H), 5.30 (d, *J* = 6.0 Hz, 1H), 5.26 (d, *J* = 6.0 Hz, 1H), 5.23 (s, 2H), 3.50 (s, 3H), 3.51 (s, 3H), 3.11 (dd, *J* = 14.2, 3.9 Hz, 1H), 2.70 (dd, *J* = 14.2, 7.1 Hz, 1H). ^13^C NMR (101 MHz, CDCl_3_) δ 200.6, 163.0, 158.7, 154.7, 133.5, 130.6, 129.0, 118.6, 115.3, 109.8, 102.9, 95.0, 94.4, 77.1, 57.0, 56.6, 40.0.

*Synthesis of 1‐(2,4‐bis(methoxymethoxy)phenyl)‐2‐hydroxy‐3‐(4‐hydroxyphenyl)propan‐1‐one 3b*. The same procedure as for (+)‐3b was used, starting from 4b (457 mg, 1.01 mmol) and obtaining 3b in 66% yield (242 mg, 0.67 mmol) as a white solid. All NMR and MS data are in agreement with the ones of the enantioenriched compound.

*Synthesis of (R)‐1‐(2,4‐bis(methoxymethoxy)phenyl)‐2‐hydroxy‐3‐(4‐hydroxy‐3‐(3‐methylbut‐2‐en‐1‐yl)phenyl)propan‐1‐one (+)‐2b*. 3b (238 mg, 0.655 mmol, 1.0 eq) was dissolved in 1.5 M aqueous KOH (0.44 mL, 0.655 mmol, 1.0 eq) at rt. 3,3‐Dimethyl allyl bromide (107 µL, 0.953 mmol, 1.4 eq) and additional 1.5 M KOH (0.44 mL, 0.655 mmol, 1.0 eq) were added in parallel, in two portions over 1 hour under vigorous stirring at 30 °C. The reaction was monitored by TLC (eluent mixture: 8:2 CH_2_Cl_2_:EtOAc, developed in potassium permanganate). After 4 hours, an additional 1.5 M KOH (0.22 mL, 0.328 mmol, 0.5 eq) was added. After 24 hours, the reaction was acidified with 1 M HCl and extracted with EtOAc (3 × 5 mL). The organic layer was dried over anhydrous Na_2_SO_4_ and concentrated under reduced pressure. The crude was purified by Biotage (eluent mixture CH_2_Cl_2_:EtOAc from 5% to 40% EtOAc) providing the pure product (+)‐2b (40 mg, 0.093 mmol, 14% yield) as a yellow oil. [α]^30^_D_ = + 73.0 (c 0.52, CHCl_3_). MS (ESI) *m/z* [M + H]^+^ = 431.2091 (calculated for C_24_H_31_O_7_, 431.2070). ^1^H NMR (400 MHz, CDCl_3_) δ 7.82 (d, *J* = 8.8 Hz, 1H), 6.91–6.84 (m, 3H), 6.78 (dd, *J* = 8.8, 2.3 Hz, 1H), 6.66 (d, *J* = 7.9 Hz, 1H), 5.35 (s, 1H), 5.31 (d, *J* = 6.8 Hz, 1H), 5.24 (d, *J* = 6.8 Hz, 1H), 5.23 (s, 2H), 3.51 (s, 3H), 3.50 (s, 4H), 3.29 (d, *J* = 7.2 Hz, 2H), 3.07 (dd, *J* = 14.2, 4.0 Hz, 1H), 2.68 (dd, *J* = 14.2, 7.1 Hz, 1H), 1.80–1.71 (m, 6H). ^13^C NMR (101 MHz, CDCl_3_) δ 200.6, 162.8, 158.5, 152.9, 134.4, 133.4, 130.9, 129.3, 128.3, 126.5, 121.9, 118.7, 115.5, 109.6, 102.8, 94.8, 94.3, 77.0, 56.8, 56.5, 40.1, 29.7, 25.8, 17.9.

*Synthesis of 1‐(2,4‐bis(methoxymethoxy)phenyl)‐2‐hydroxy‐3‐(4‐hydroxy‐3‐(3‐methylbut‐2‐en‐1‐yl)phenyl)propan‐1‐one 2b*. The same procedure as for (+)‐2b was used, starting from 3b (242 mg, 0.668 mmol) and obtaining 2b in 14% yield (41 mg, 0.094 mmol) as a yellow oil. All NMR and MS data are in agreement with the ones of the enantioenriched compound.

*Synthesis of glycybridin B (−)‐1*. MOM‐protected glycybridin B (+)‐2b (20 mg, 0.046 mmol, 1 eq) was dissolved in MeOH (3 mL). Activated Dowex 50 × 8 was added under stirring, and the mixture was stirred at rt for 48 hours, monitoring the conversion via HPLC (sampling 50 µL every 24 hours, filtering it, diluting with 1 mL of MeOH, and analyzing it on a KROMAPHASE 100 C18 5 µm 150 × 4.6 mm with H_2_O/ACN from 90:10 to 0:100 in 30 minutes, flow rate 1 mL/min, product tr = 15.9 minutes). Then, the reaction mixture was filtered and concentrated under reduced pressure. The crude was purified via flash chromatography (eluent mixture: 98:2 to 86:14 CH_2_Cl_2_:EtOAc) and the product was recovered (3.6 mg, 0.008 mmol, 23% yield, ee% = 89%) as a white solid. [α]^30^_D_ = ‐14.7 (c 0.1, CHCl_3_). HRMS (ESI) *m/z* [M‐H]^−^ = 341.1399 (calculated for C_20_H_21_O_5_, 341.1389). ^1^H NMR (400 MHz, CDCl_3_) δ 12.02 (s, 1H), 7.54 (d, *J* = 8.6 Hz, 1H), 6.87 (dd, *J* = 8.2, 2.3 Hz, 1H), 6.82 (d, *J* = 2.3 Hz, 1H), 6.70 (d, *J* = 8.1 Hz, 1H), 6.44–6.36 (m, 2H), 6.01 (s, 1H), 5.29–5.16 (m, 2H), 5.06 (s, 1H), 3.56 (s, 2H), 3.28 (d, *J* = 7.2 Hz, 2H), 3.09 (dd, *J* = 14.2, 4.5 Hz, 1H), 2.90 (dd, *J* = 14.2, 6.5 Hz, 1H), 1.75 (s, 6H). ^13^C NMR (101 MHz, CDCl_3_) δ 203.2, 165.7, 163.5, 153.5, 135.0, 132.0, 131.2, 128.6, 128.2, 127.0, 121.8, 115.9, 111.3, 108.5, 104.0, 77.5, 77.2, 76.9, 72.9, 42.4, 29.9, 25.9, 18.0.

*Synthesis of glycybridin B 1*. The same procedure as for *(−)‐*1 was used, starting from 2b (41 mg, 0.096 mmol) and obtaining 1 in 20% yield (6.5 mg, 0.019 mmol) as a white solid. All NMR and MS data are in agreement with the ones of the enantioenriched compound.

### 4.3 Biological Evaluation

#### 4.3.1 Inhibition of Tubulin Assembly

Tubulin polymerization was followed by time course fluorimetry at λ_abs_ = 350 nm, λ_em_ = 350 nm in 384‐well black polystyrene plates (Greiner) that were kept at 37 °C throughout the experiment and read in a PHERAstar FSX plate reader (BMG Labtech). Kinetics were obtained from triplicate reactions for each condition. Experimental curves were averaged and normalized by subtraction of the minimum absorbance value. The assays were carried out employing 15 µM tubulin in BRB80 buffer (80 mM Pipes‐KOH, 1 mM MgCl_2_, 1 mM EGTA, 1 mM GTP) with 10 µM DAPI, 3.4 M glycerol, and 1 mM GTP. 30 or 40 µM of each glycybridine B derivative was added to the reaction mixture prior to polymerization. 40 µM Nocodazole and an equivalent volume of DMSO were employed as controls. The mixtures were kept at 4 °C upon addition to the plate to avoid tubulin polymerization or aggregation before the plate measurement started.

## Conflict of Interest

The authors declare no conflict of interest.

## Supporting information



Supplementary Information

## Data Availability

The data that support the findings of this study are available in the supplementary material of this article.

## References

[1] H. V. Goodson , E. M. Jonasson , Cold Spring Harb. Perspect. Biol. 2018, 10, a022608. 29858272 10.1101/cshperspect.a022608 PMC5983186

[2] M. O. Steinmetz , A. E. Prota , Trends Cell Biol. 2018, 28, 776. 29871823 10.1016/j.tcb.2018.05.001

[3] V. Čermák , V. Dostál , M. Jelínek , L. Libusová , J. Kovář , D. Rösel , J. Brábek , Eur. J. Cell Biol. 2020, 99, 151075. 32414588 10.1016/j.ejcb.2020.151075

[4] Z. Boiarska , H. Pérez‐Peña , A.‐C. Abel , P. Marzullo , B. Álvarez‐Bernad , F. Bonato , B. Santini , D. Horvath , D. Lucena‐Agell , F. Vasile , M. Sironi , J. Fernando Díaz , A. E. Prota , S. Pieraccini , D. Passarella , Chem. Eur. J. 2022, 29, e202203431. 36468686 10.1002/chem.202203431

[5] K. Li , S. Ji , W. Song , Y. Kuang , Y. Lin , S. Tang , Z. Cui , X. Qiao , S. Yu , M. Ye , A.‐K. Glycybridins , J. Nat. Prod. 2017, 80, 334. 28140583 10.1021/acs.jnatprod.6b00783

[6] C. Li , T. Eom , Y. Jeong , J. Nutr. Sci. Vitaminol. (Tokyo) 2015, 61, 375. 26639845 10.3177/jnsv.61.375

[7] D. Dhingra , A. Sharma , Prog. Neuropsychopharmacol. Biol. Psychiatry 2006, 30, 449. 16443316 10.1016/j.pnpbp.2005.11.019

[8] G. Wolber , T. Langer , J. Chem. Inf. Model. 2005, 45, 160. 15667141 10.1021/ci049885e

[9] B. Zdrazil , E. Felix , F. Hunter , E. J. Manners , J. Blackshaw , S. Corbett , M. de Veij , H. Ioannidis , D. M. Lopez , J. F. Mosquera , M. P. Magarinos , N. Bosc , R. Arcila , T. Kizilören , A. Gaulton , A. P. Bento , M. F. Adasme , P. Monecke , G. A. Landrum , A. R. Leach , Nucleic Acids Res. 2024, 52, D1180. 37933841 10.1093/nar/gkad1004 PMC10767899

[10] O. Korb , T. Stützle , T. E. Exner , PLANTS: Application of Ant Colony Optimization to Structure‐Based Drug Design. In: M. Dorigo , L. M. Gambardella , M. Birattari , A. Martinoli , R. Poli , T. Stützle , (eds) Ant Colony Optimization and Swarm Intelligence. ANTS 2006. Lecture Notes in Computer Science, Vol. 4150. Springer, Berlin, Heidelberg.

[11] R. M. Moriarty , S. Grubjesic , B. C. Surve , S. N. Chandersekera , O. Prakash , R. Naithani , Eur. J. Med. Chem. 2006, 41, 263. 16330130 10.1016/j.ejmech.2005.09.008

[12] E. J. Corey , F. Y. Zhang , Org. Lett. 1999, 1, 1287. 10825978 10.1021/ol990964z

[13] A. Lattanzi , Adv. Synth. Catal. 2006, 348, 339.

[14] K. M. Weiß , S. B. Tsogoeva , Chem. Rec. 2011, 11, 18. 21308970 10.1002/tcr.201000006

[15] A. Gerlach , T. Geller , Adv. Synth. Catal. 2004, 346, 1247.

[16] A. Gotoh , M. Hidaka , K. Hirose , T. Uchida , J. Biol. Chem. 2013, 288, 34699. 24151073 10.1074/jbc.M113.513119 PMC3843081

[17] C. Heusele , D. Bonne , C. Simon , D. Pantaloni , EJB 1987, 165, 613.3595603

[18] D. Bonne , C. Heusele , C. Simon , D. Pantaloni , JBC 1985, 260, 2819. 3972806

[19] C. E. Meng , T. D. Goddard , E. F. Pettersen , G. S. Couch , Z. J. Pearson , J. H. Morris , T. E. Ferrin , Protein Sci. 2023, 32, e4792. 37774136 10.1002/pro.4792 PMC10588335

[20] P. A. de Almeida , S. V. Fraiz Jr. , R. Braz‐Filho , J. Braz. Chem. Soc. 1999, 10, 347.

[21] D. R. Brandt , K. M. Pannone , J. J. Romano , E. G. Casillas , Tetrahedron 2013, 69, 9994.

