# Supplementary material for: Asymmetric Total Synthesis of (−)‐Glycybridin B, a Pharmacophore Screened Candidate for Tubulin Binding
Source: Chemistry. 2025 Aug 6;31(55):e02228. doi: 10.1002/chem.202502228 (PMC12498065; doi:10.1002/chem.202502228)

Chemistry—A European Journal

Supporting Information

Asymmetric Total Synthesis of (-)-Glycybridin B, a Pharmacophore Screened Candidate for Tubulin Binding

Alice Maiocchi,[a] Maxim Shevelev,[b] Zlata Boiarska,[a] Juan Estévez-Gallego,[c] Francesca Bonato,[a] Paolo Orlando,[a] Alessandra Chinosi,[a] Emanuele Marcone,[a] Andrea Citarella,[a] Dragos Horvath,[b] Michel O. Steinmetz,[c],[d] Andrea E. Prota,[c] Alexandre Varnek,[b] Valerio Fasano,*[a] and Daniele Passarella*[a]

NMR spectra

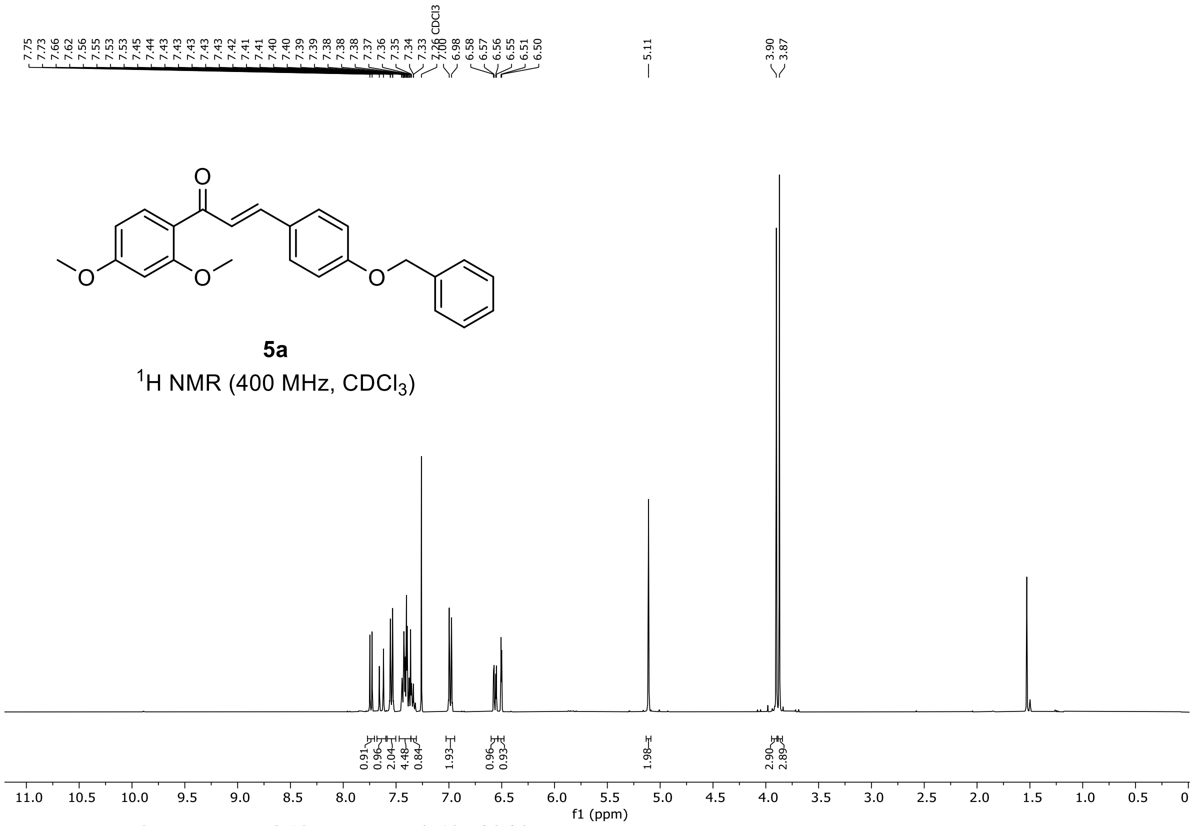
5a
^{1}H NMR (400 MHz, $CDCl_3$)
7.75 7.73 7.66 7.62 7.56 7.55 7.53 7.53 7.45 7.44 7.43 7.43 7.43 7.43 7.42 7.41 7.41 7.40 7.40 7.39 7.39 7.38 7.38 7.38 7.37 7.36 7.35 7.34 7.33 7.26 CDCl3 7.00 6.98 6.58 6.57 6.56 6.55 6.51 6.50
5.11
3.90 3.87
f1 (ppm)

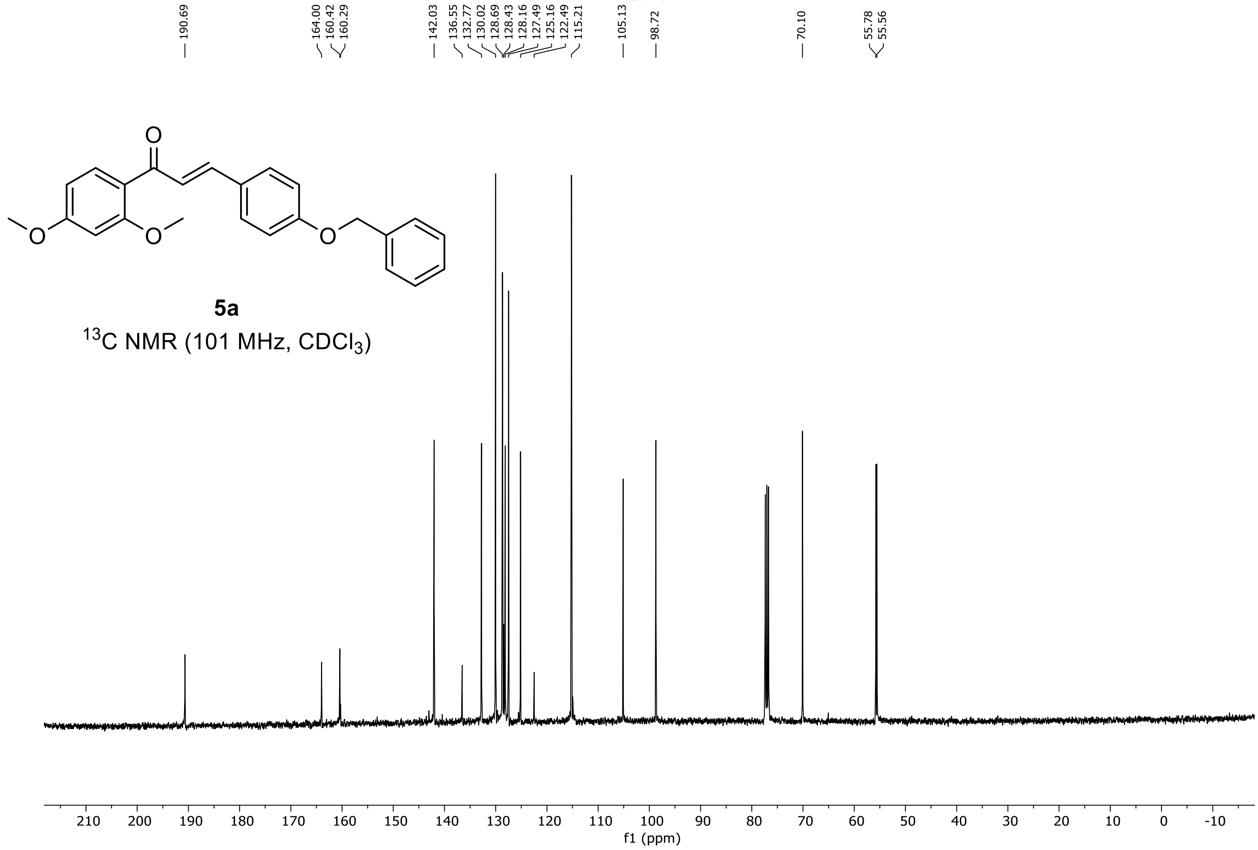
5a
^{13}C NMR (101 MHz, $CDCl_3$)
190.69
164.00 160.42 160.29
142.03 136.55 132.77 130.02 128.69 128.43 128.16 127.49 125.16 122.49 115.21
105.13 98.72
70.10
55.78 55.56
f1 (ppm)

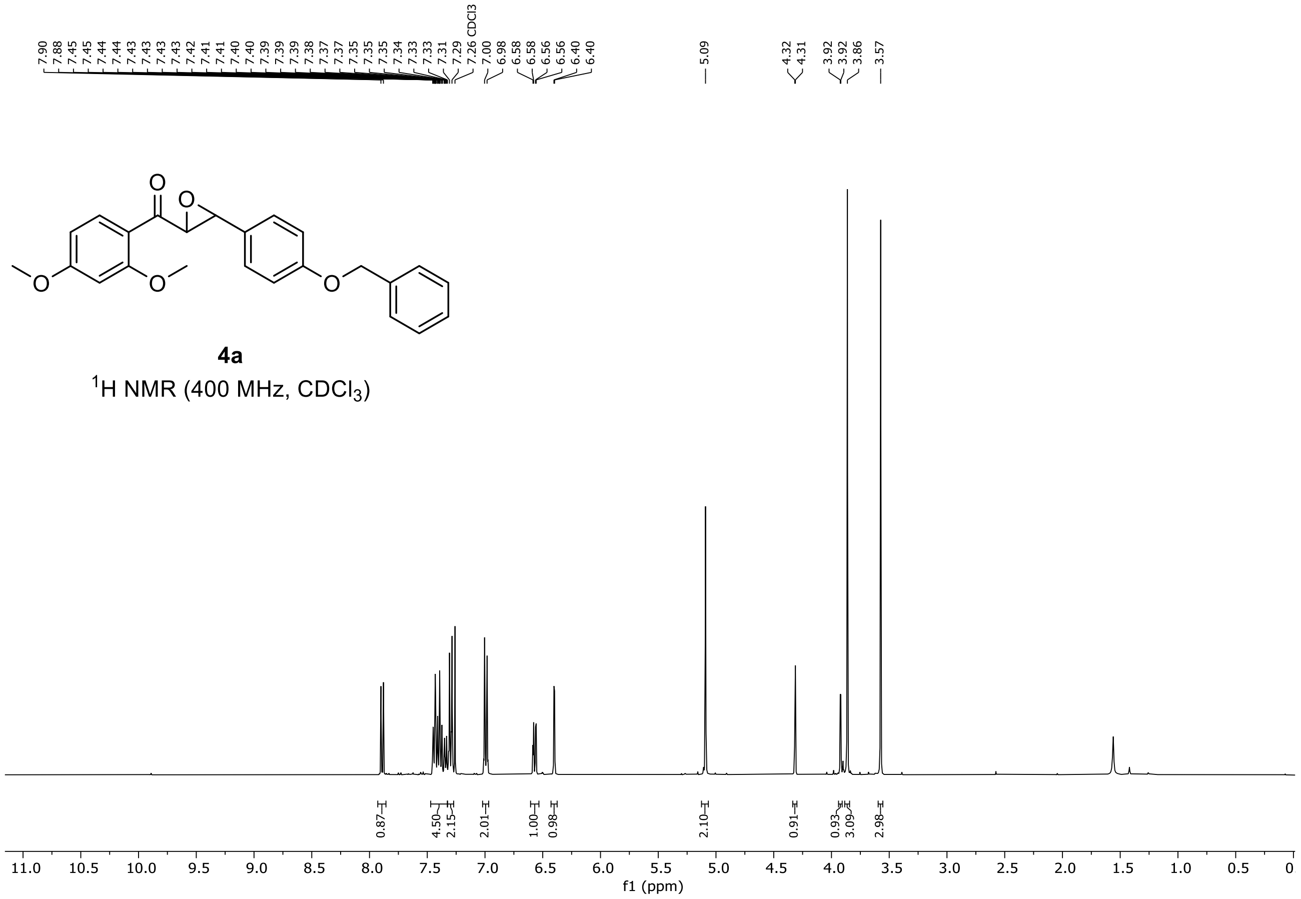

4a

^{1}H NMR (400 MHz, $CDCl_3$)

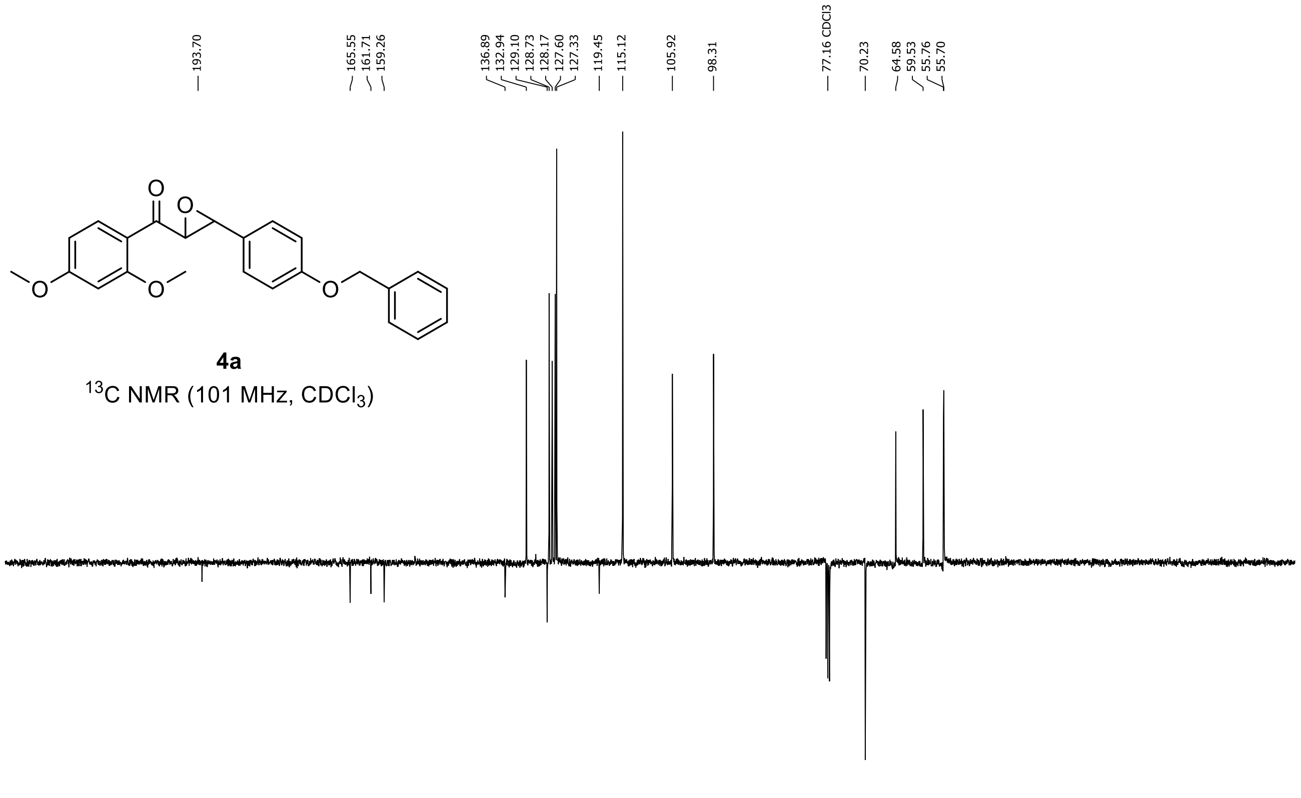

4a

^{13}C NMR (101 MHz, $CDCl_3$)

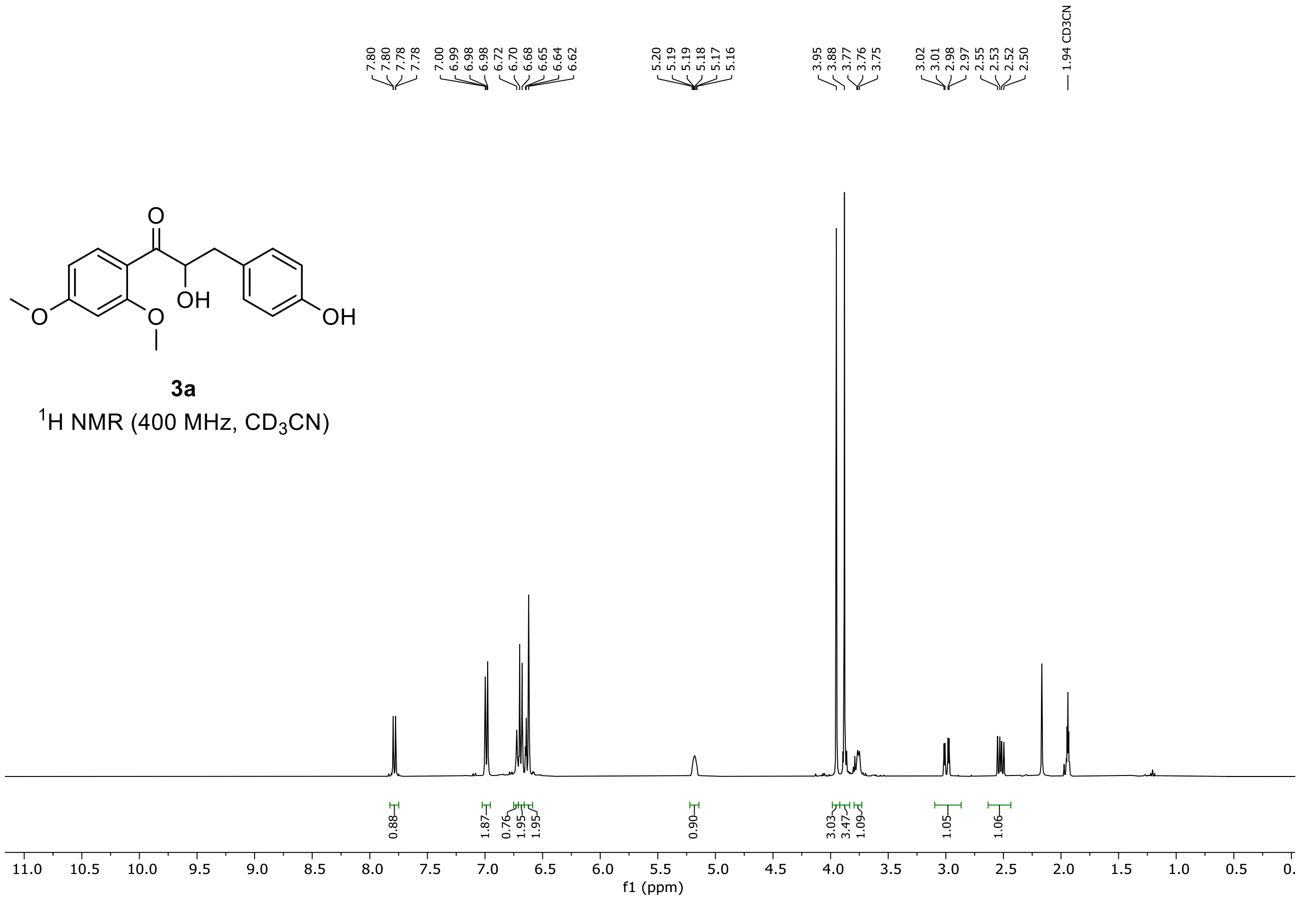

3a

^{1}H NMR (400 MHz, CD_3CN)

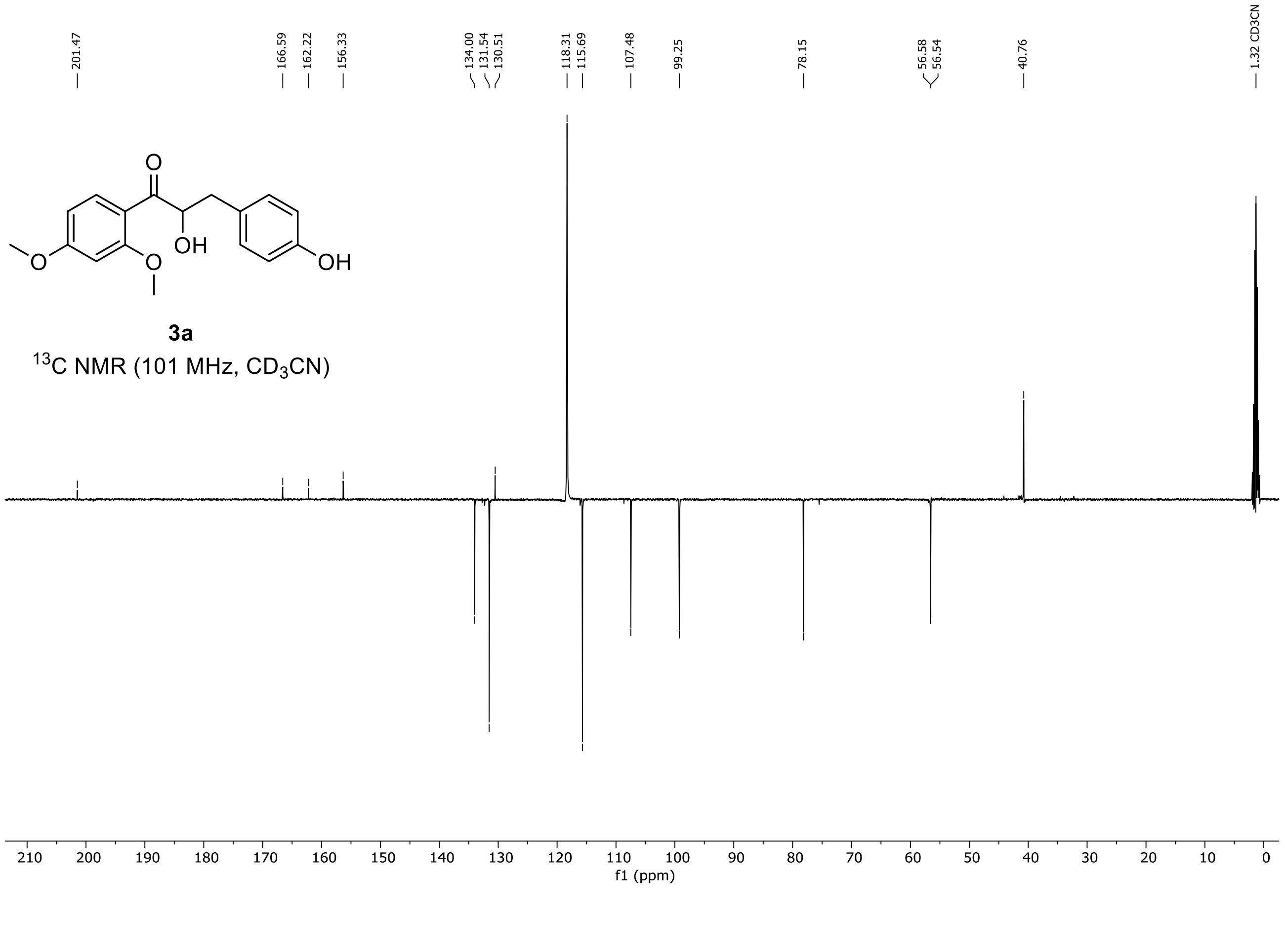

3a

^{13}C NMR (101 MHz, CD_3CN)

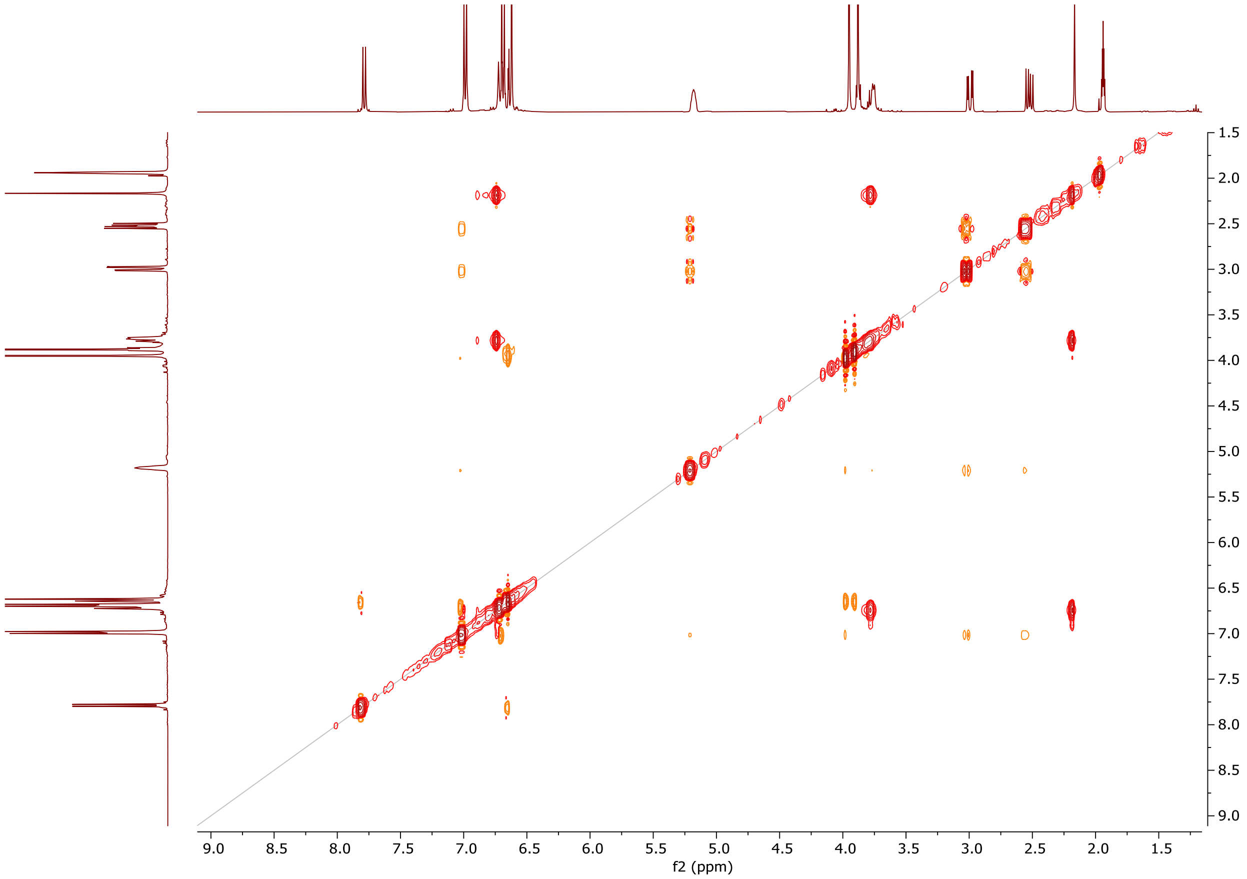

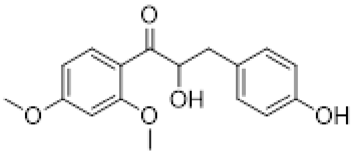

3a NOESY

The NOESY experiment shows an interaction between the signal at 5.18 ppm, attributed to the hydrogen on the C–OH carbon, and the signal at 3.95 ppm, which corresponds to one of the two MeO groups (presumably the one in the ortho position). The CH_2 groups do not show any interaction with the MeO signals.

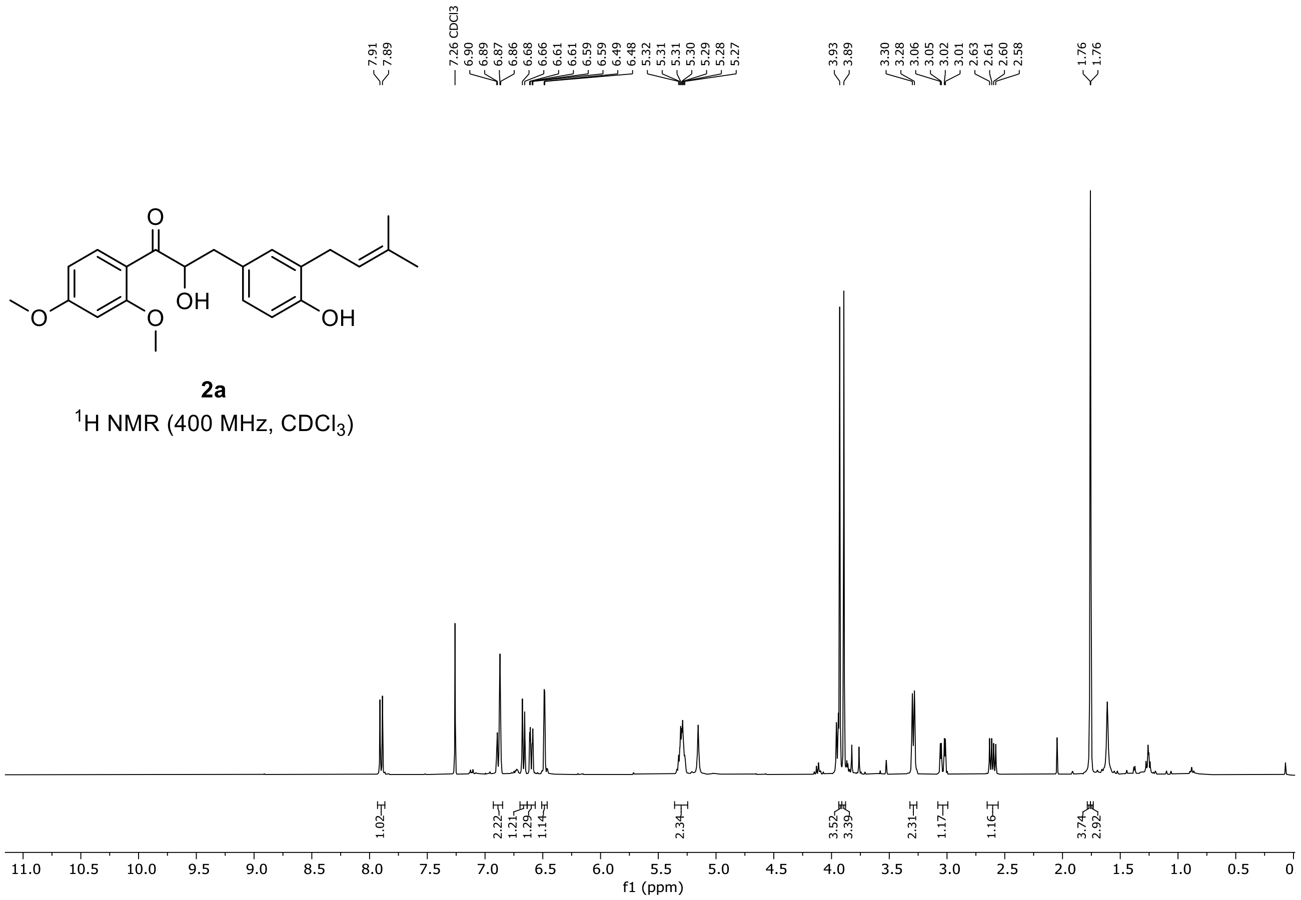

7.91
7.89
7.26 CDCl3
6.90
6.89
6.87
6.86
6.68
6.66
6.61
6.61
6.59
6.59
6.49
6.48
5.32
5.31
5.31
5.30
5.29
5.28
5.27
3.93
3.89
3.30
3.28
3.06
3.05
3.02
3.01
2.63
2.61
2.60
2.58
1.76
1.76
OH
OH
O
2a
^{1}H NMR (400 MHz, CDCl$_3$)
1.02
2.22
1.21
1.29
1.14
2.34
3.52
3.39
2.31
1.17
1.16
3.74
2.92
f1 (ppm)

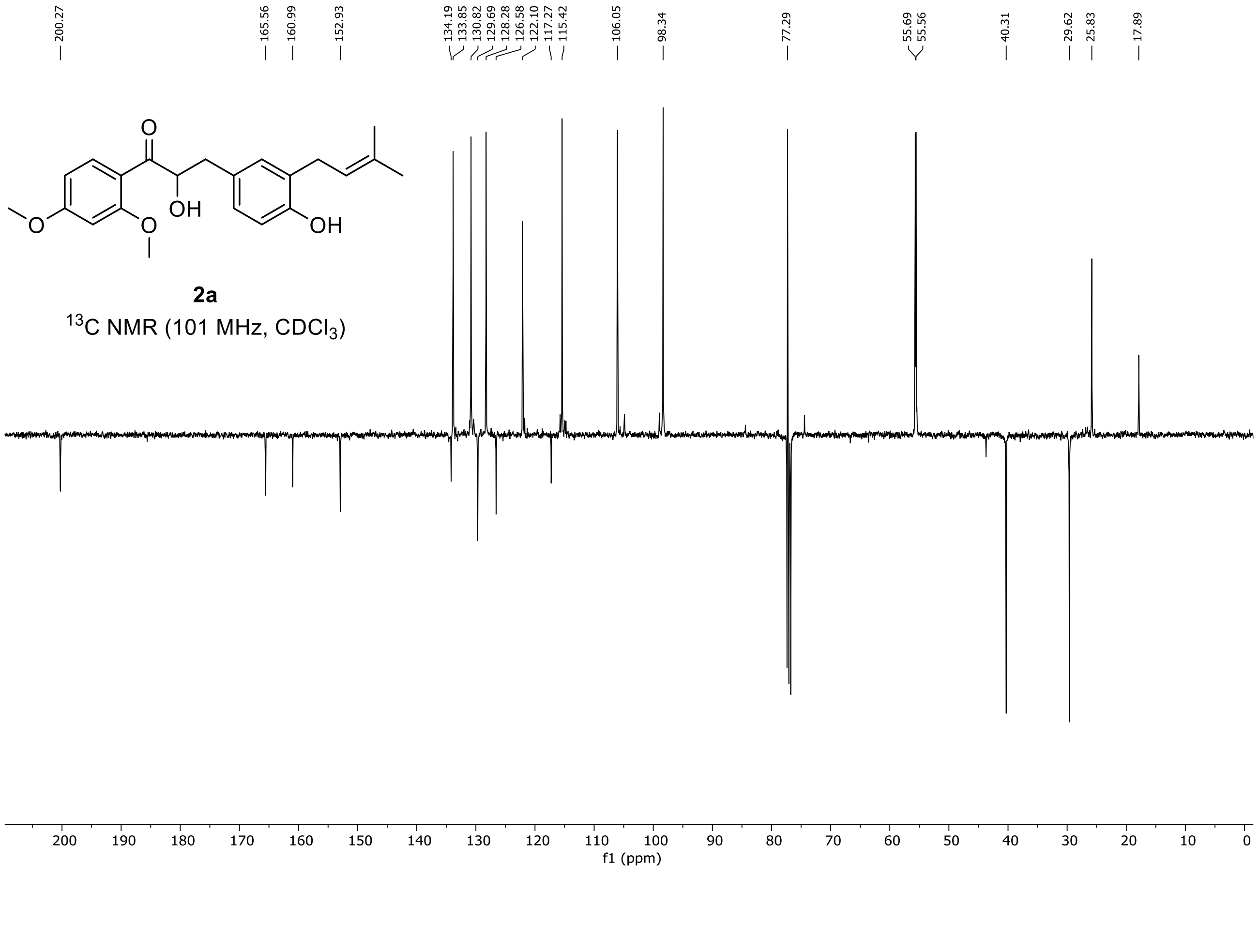

200.27
165.56
160.99
152.93
134.19
133.85
130.82
129.69
128.28
126.58
122.10
117.27
115.42
106.05
98.34
77.29
55.69
55.56
40.31
29.62
25.83
17.89
OH
OH
O
2a
^{13}C NMR (101 MHz, CDCl$_3$)
f1 (ppm)

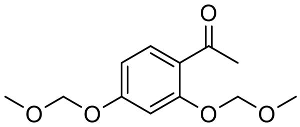

6b

^{1}H NMR (400 MHz, $CDCl_3$)

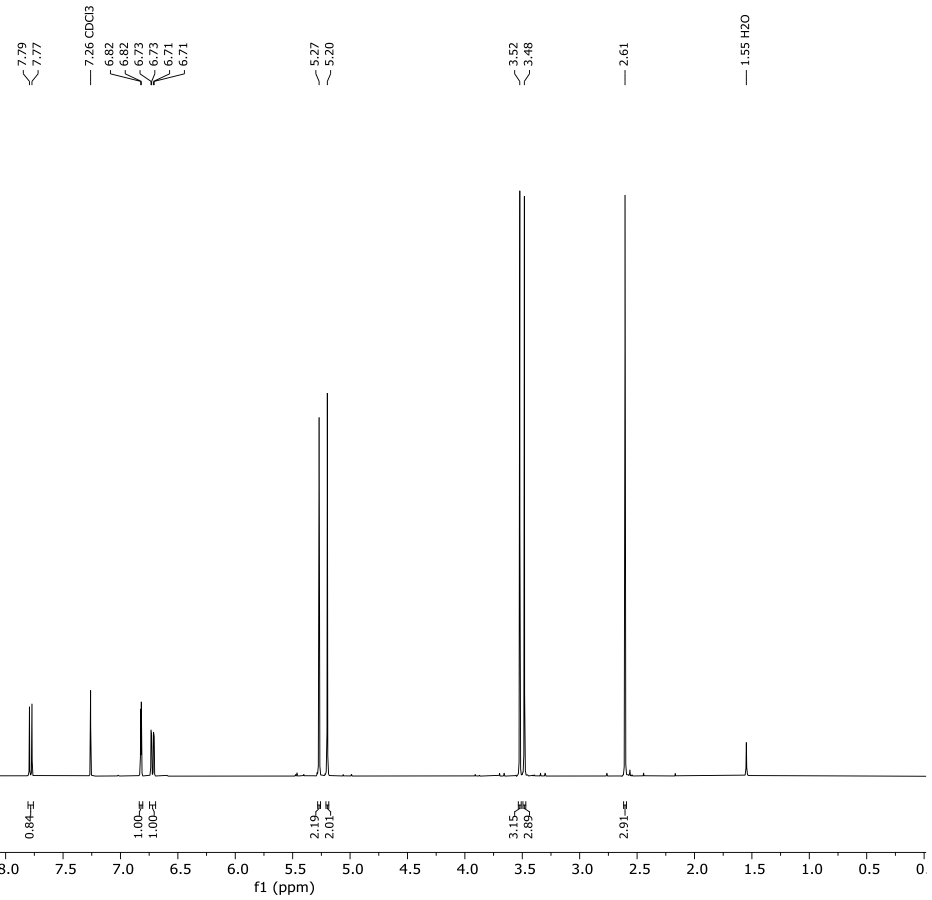

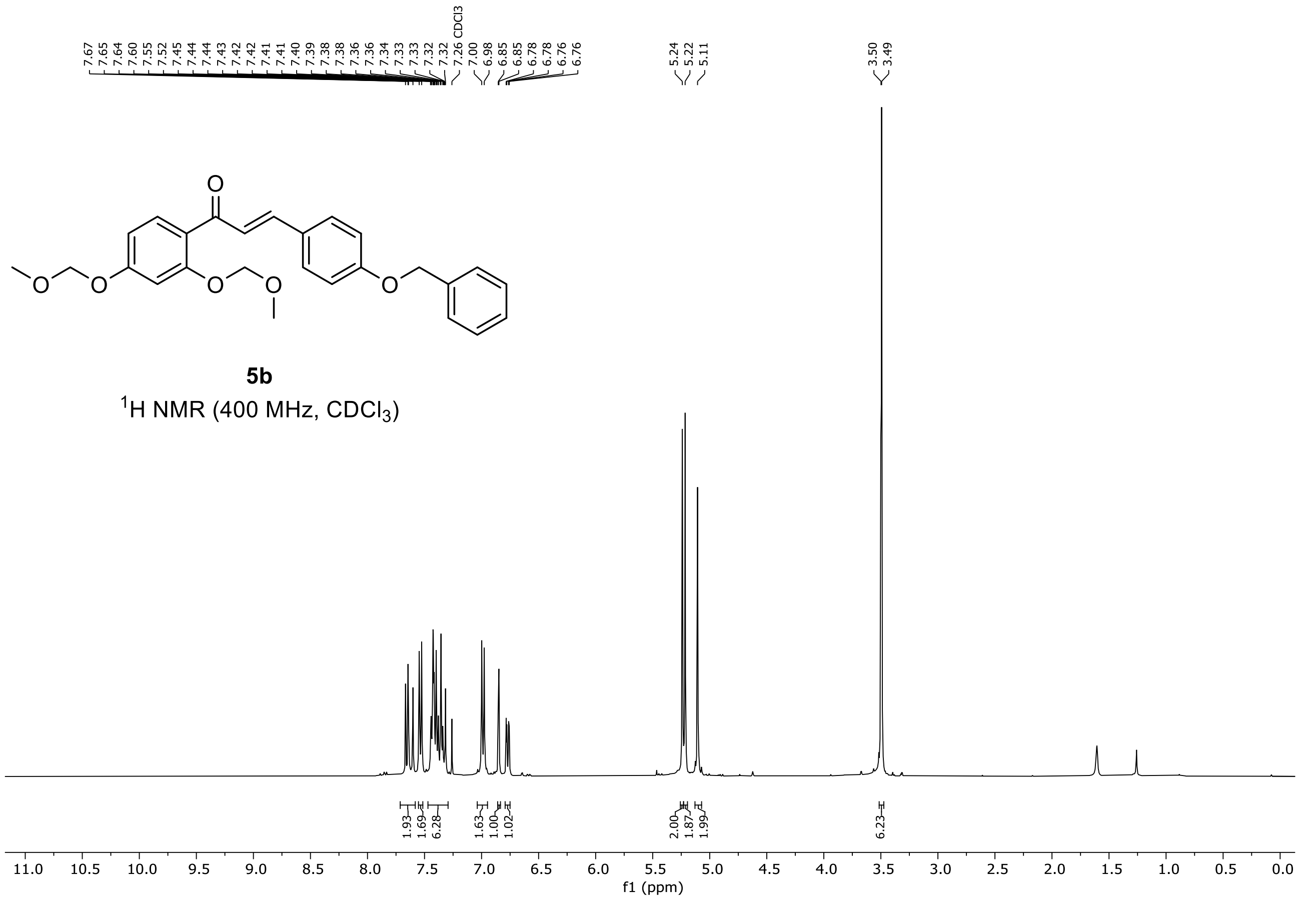
7.67
7.65
7.64
7.60
7.55
7.52
7.45
7.44
7.44
7.43
7.42
7.42
7.41
7.41
7.40
7.39
7.38
7.38
7.36
7.36
7.34
7.33
7.33
7.32
7.32
7.26 CDCl3
7.00
6.98
6.85
6.85
6.78
6.78
6.76
6.76
5.24
5.22
5.11
3.50
3.49
O
O
O
O
O
O
5b
^{1}H NMR (400 MHz, $CDCl_3$)
1.93
1.69
6.28
1.63
1.00
1.02
2.00
1.87
1.99
6.23
11.0 10.5 10.0 9.5 9.0 8.5 8.0 7.5 7.0 6.5 6.0 5.5 5.0 4.5 4.0 3.5 3.0 2.5 2.0 1.5 1.0 0.5 0.0
f1 (ppm)

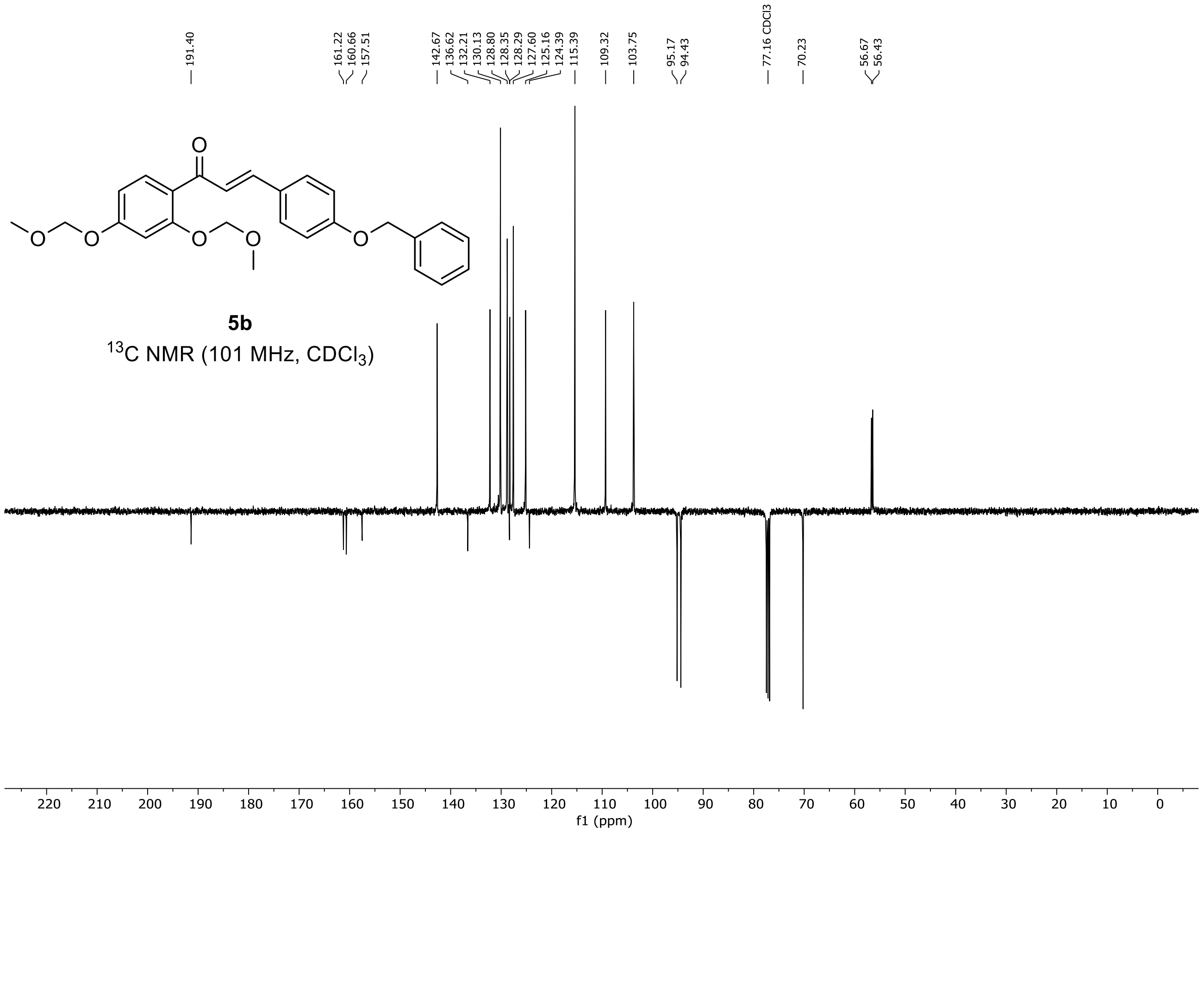
191.40
161.22
160.66
157.51
142.67
136.62
132.21
130.13
128.80
128.35
128.29
127.60
125.16
124.39
115.39
109.32
103.75
95.17
94.43
77.16 CDCl3
70.23
56.67
56.43
O
O
O
O
O
O
5b
^{13}C NMR (101 MHz, $CDCl_3$)
220 210 200 190 180 170 160 150 140 130 120 110 100 90 80 70 60 50 40 30 20 10 0
f1 (ppm)

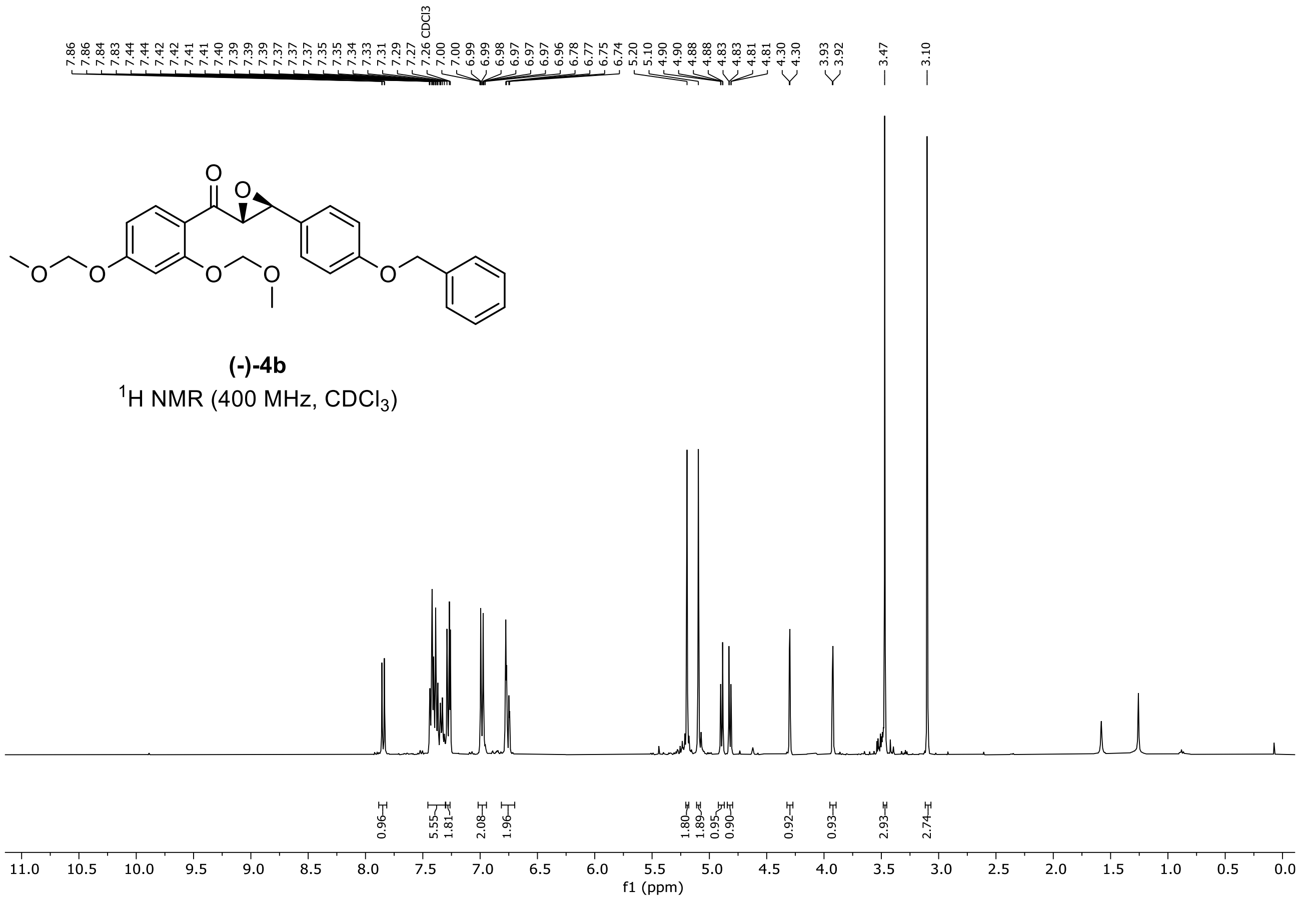

(-)-4b
^{1}H NMR (400 MHz, $CDCl_3$)

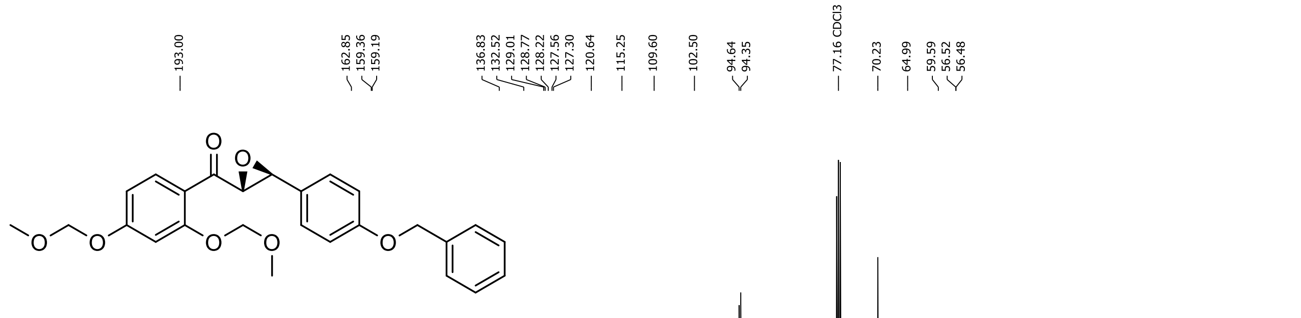

(-)-4b
^{13}C NMR (101 MHz, $CDCl_3$)

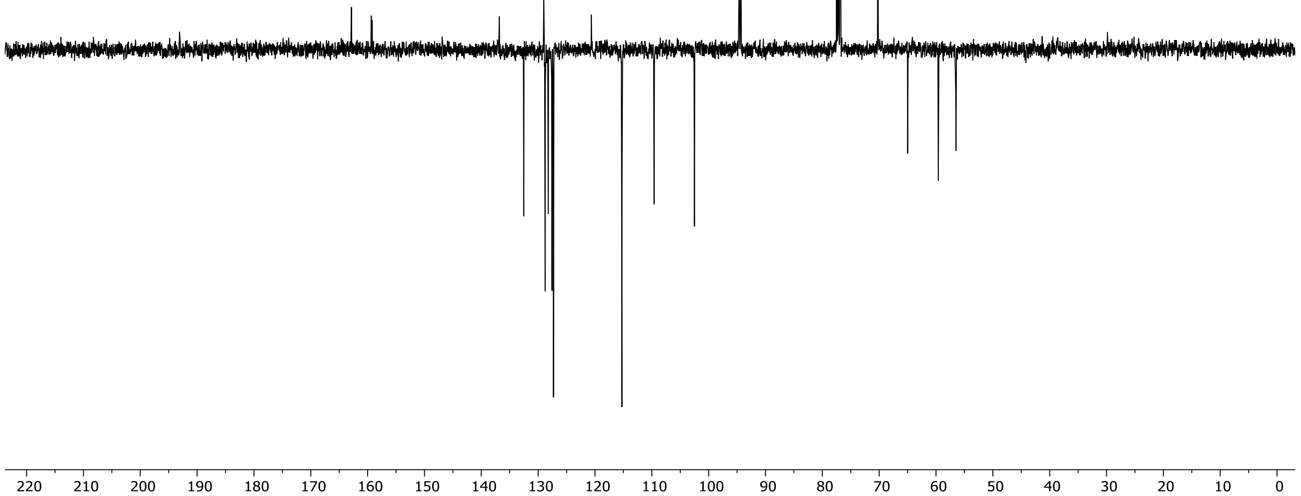

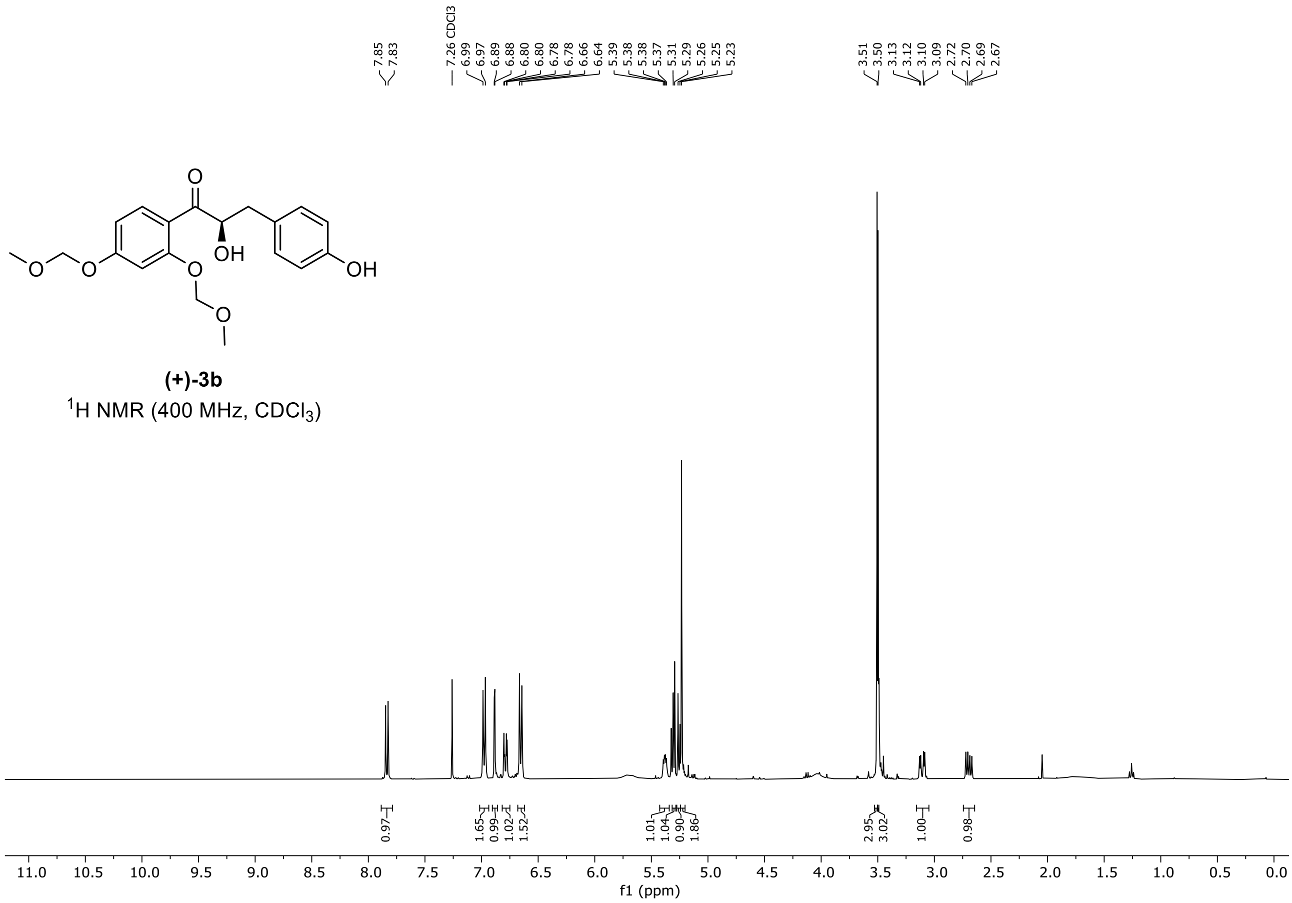

(+)-3b

^{1}H NMR (400 MHz, $CDCl_3$)

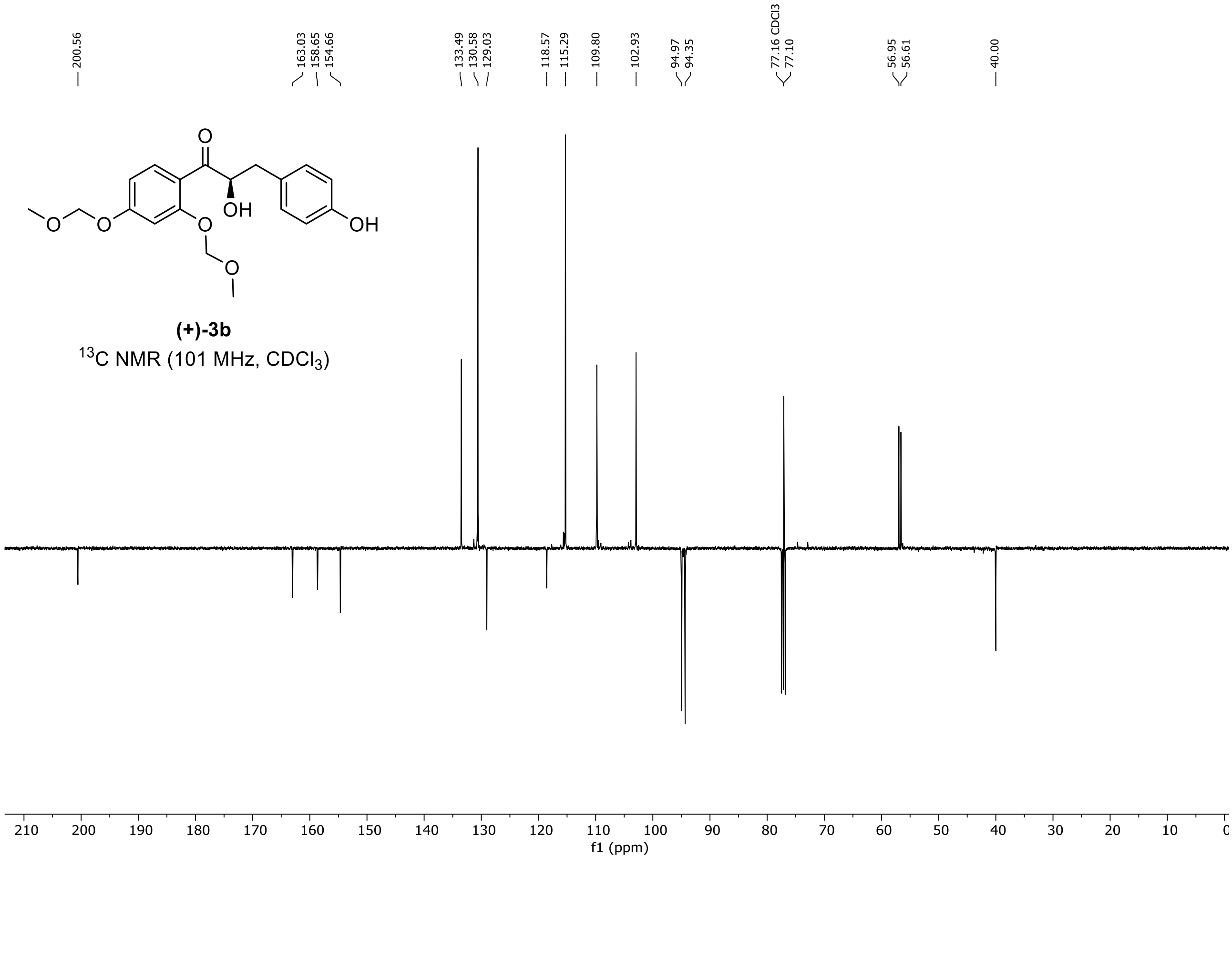

(+)-3b

^{13}C NMR (101 MHz, $CDCl_3$)

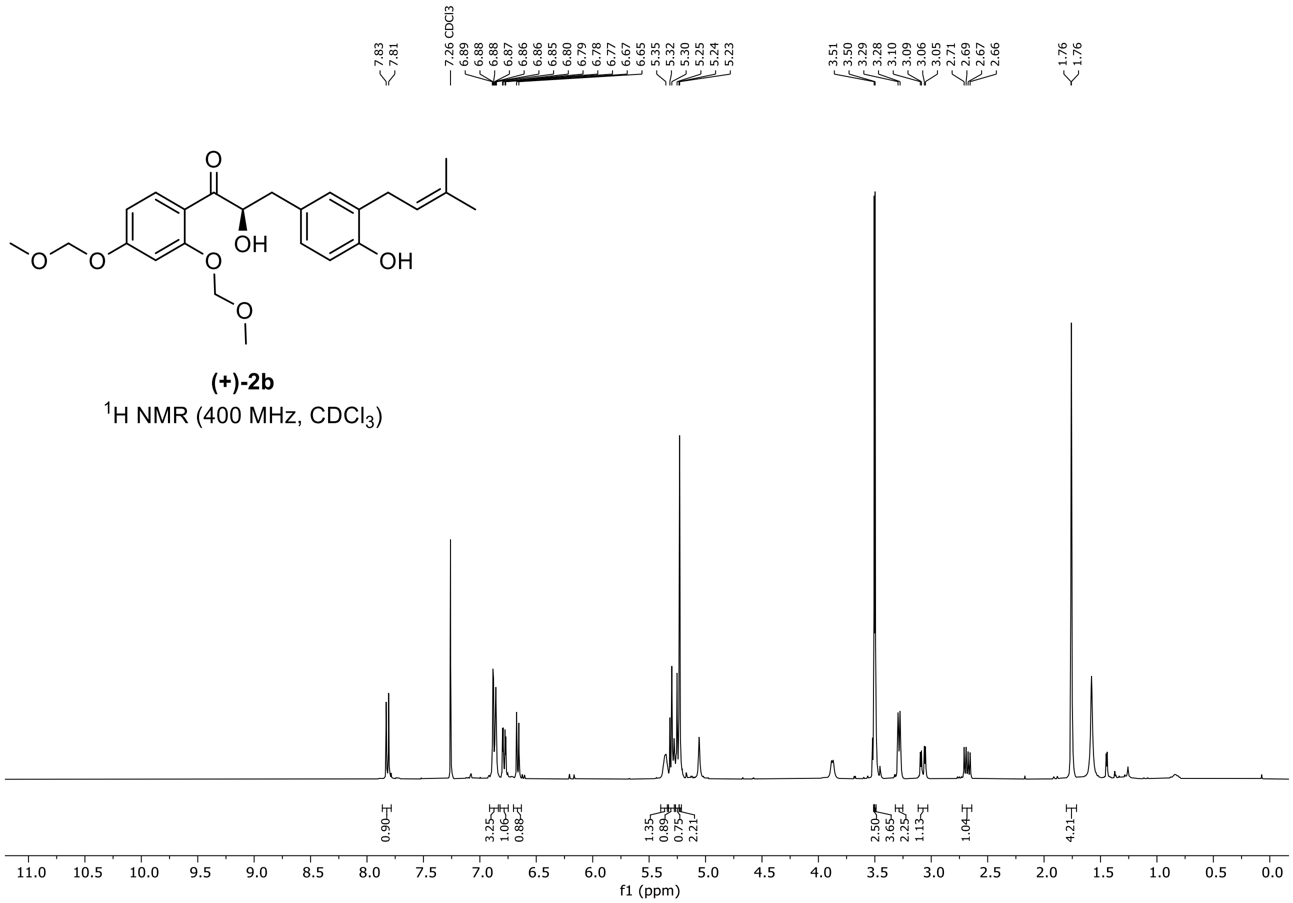

(+)-2b

^{1}H NMR (400 MHz, $CDCl_3$)

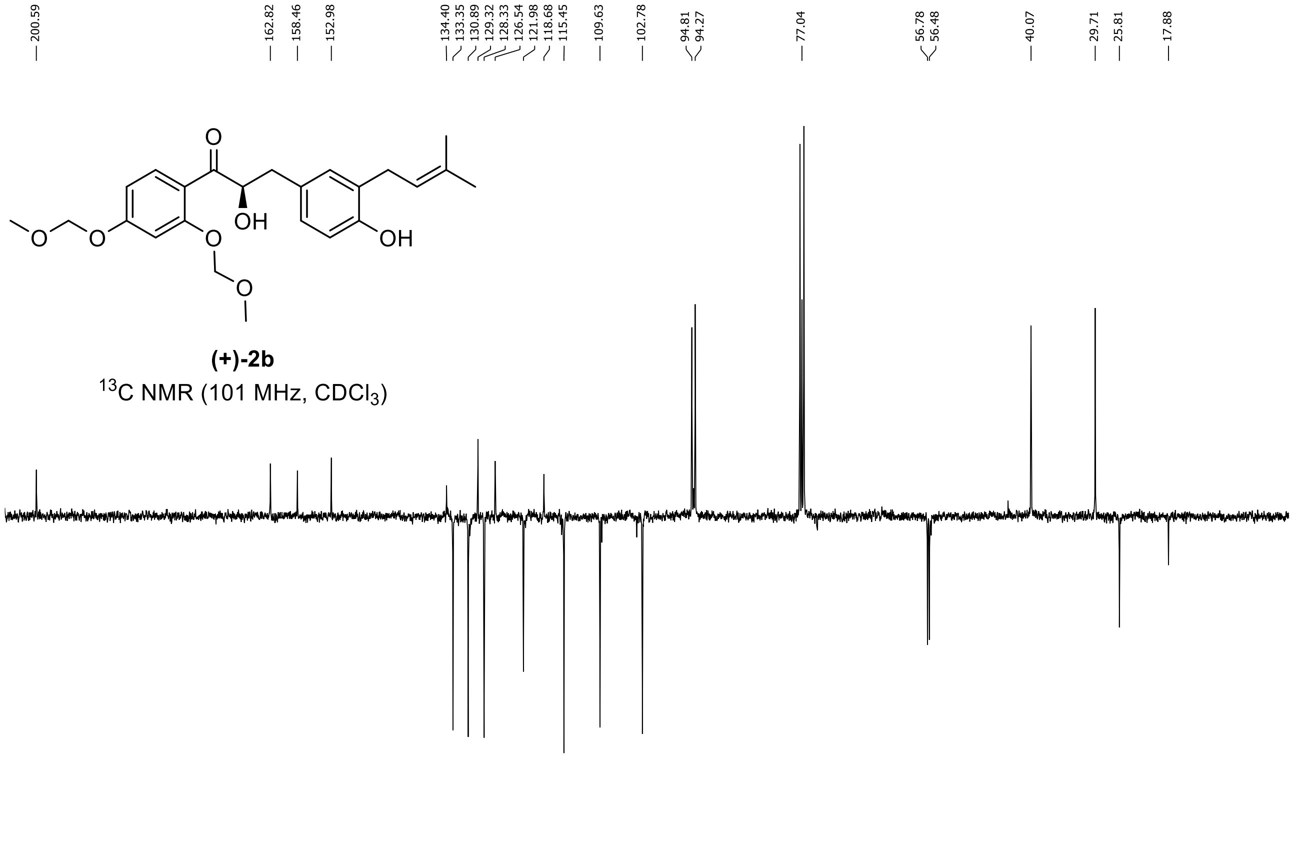

(+)-2b

^{13}C NMR (101 MHz, $CDCl_3$)

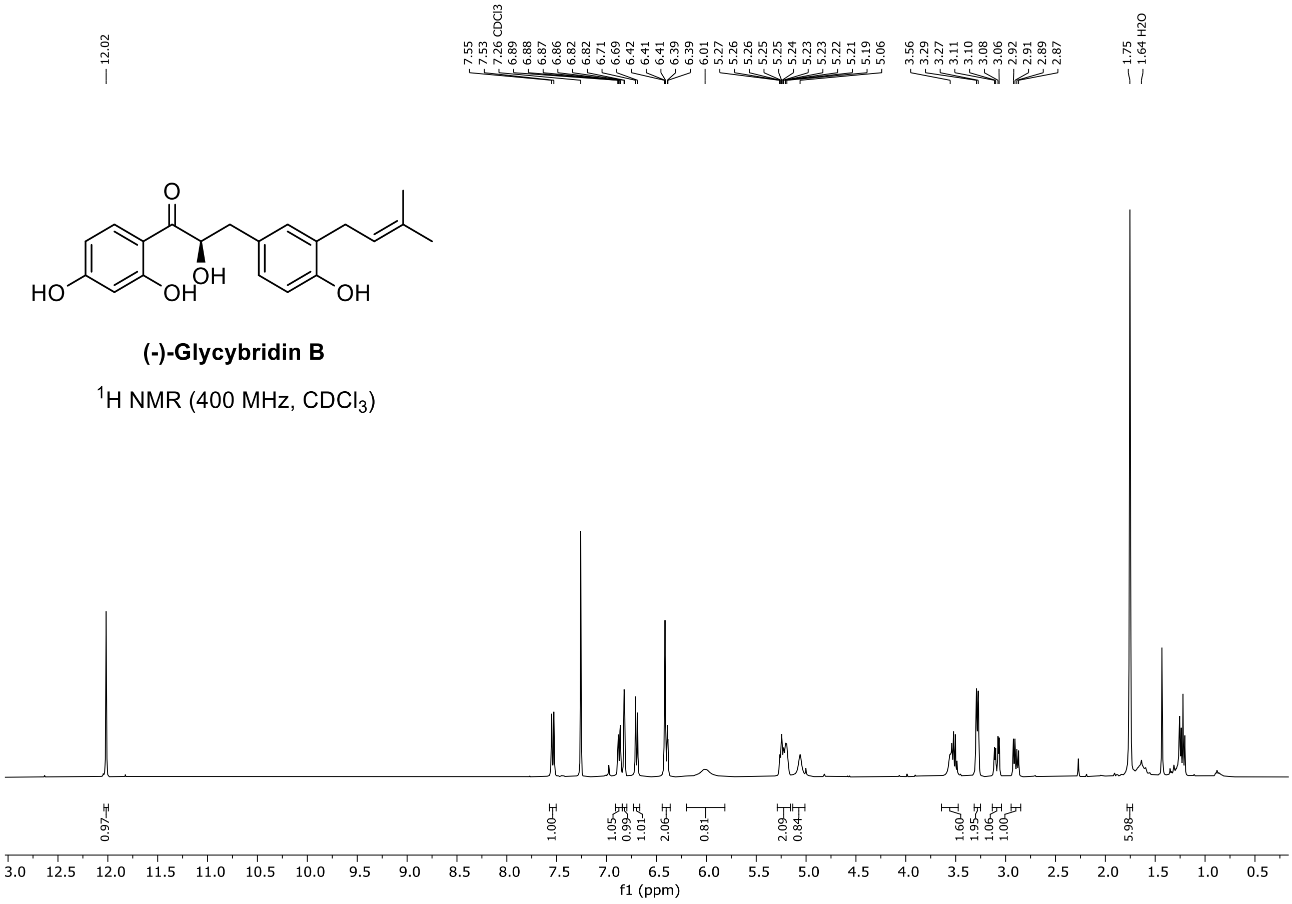

(-)-Glycybridin B

^{1}H NMR (400 MHz, $CDCl_3$)

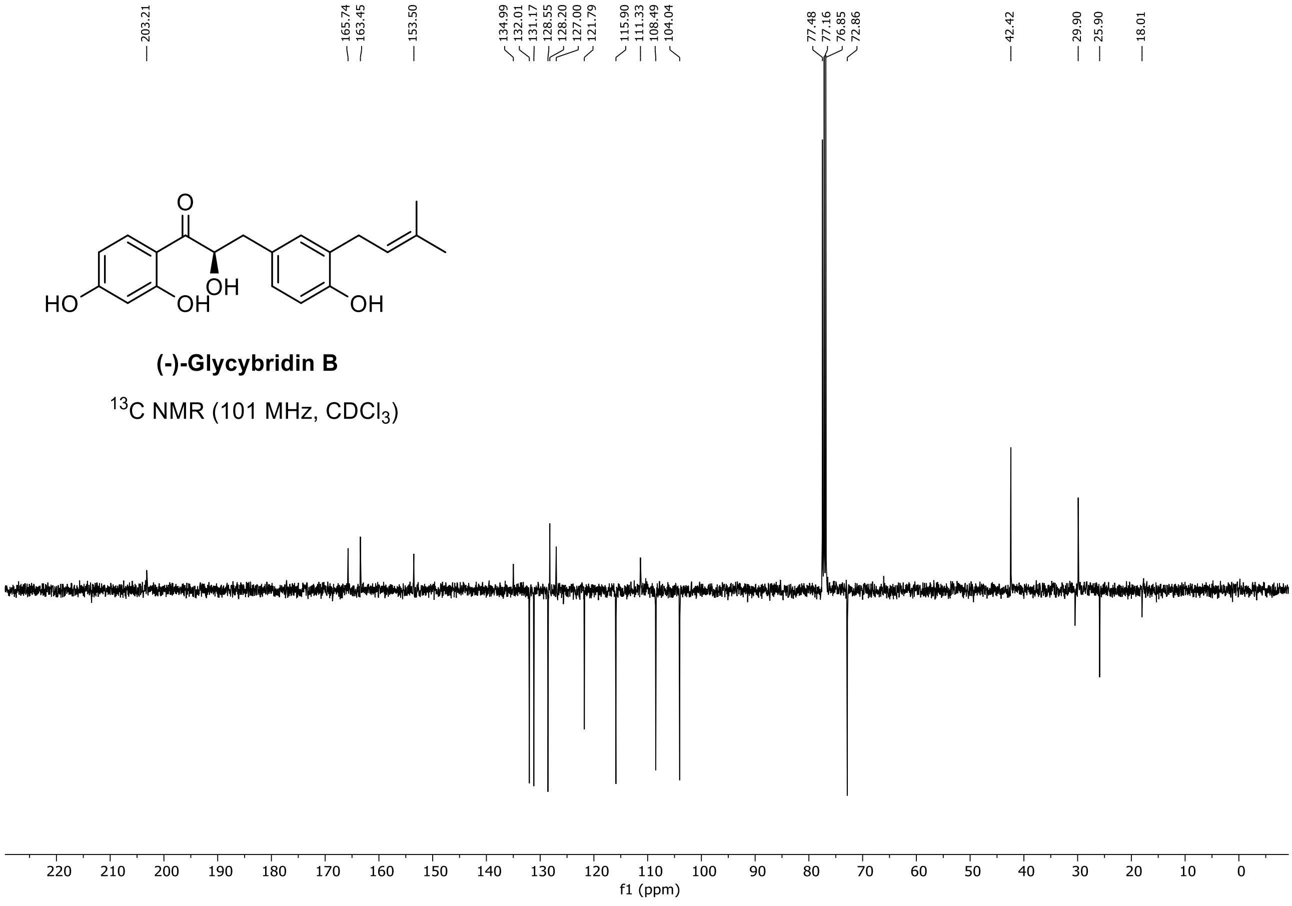

(-)-Glycybridin B

^{13}C NMR (101 MHz, $CDCl_3$)

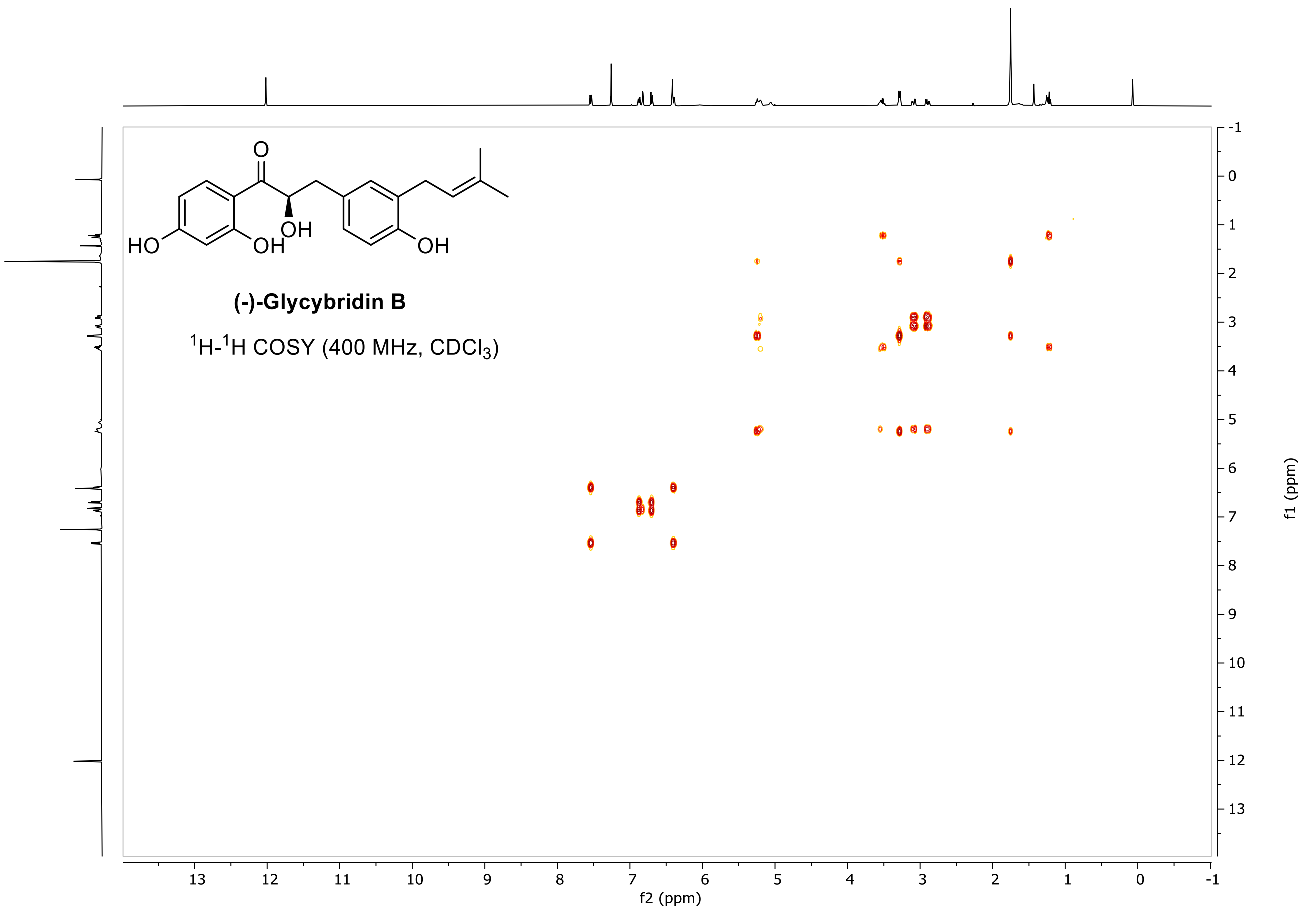

HO
OH
OH
OH
O
(-)-Glycybridin B
^{1}H-^{1}H COSY (400 MHz, CDCl$_3$)
f2 (ppm)
f1 (ppm)

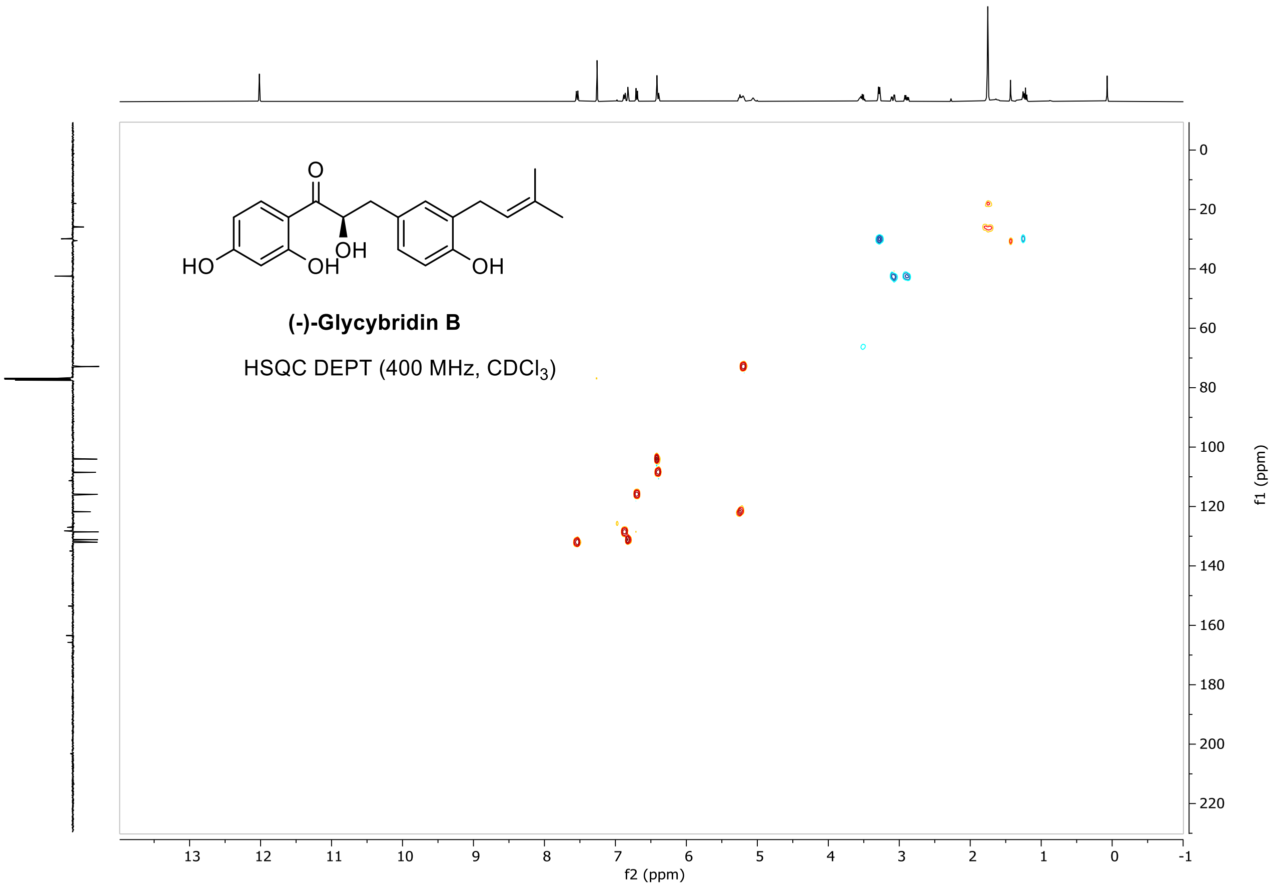

HO
OH
OH
OH
O
(-)-Glycybridin B
HSQC DEPT (400 MHz, CDCl$_3$)
f2 (ppm)
f1 (ppm)

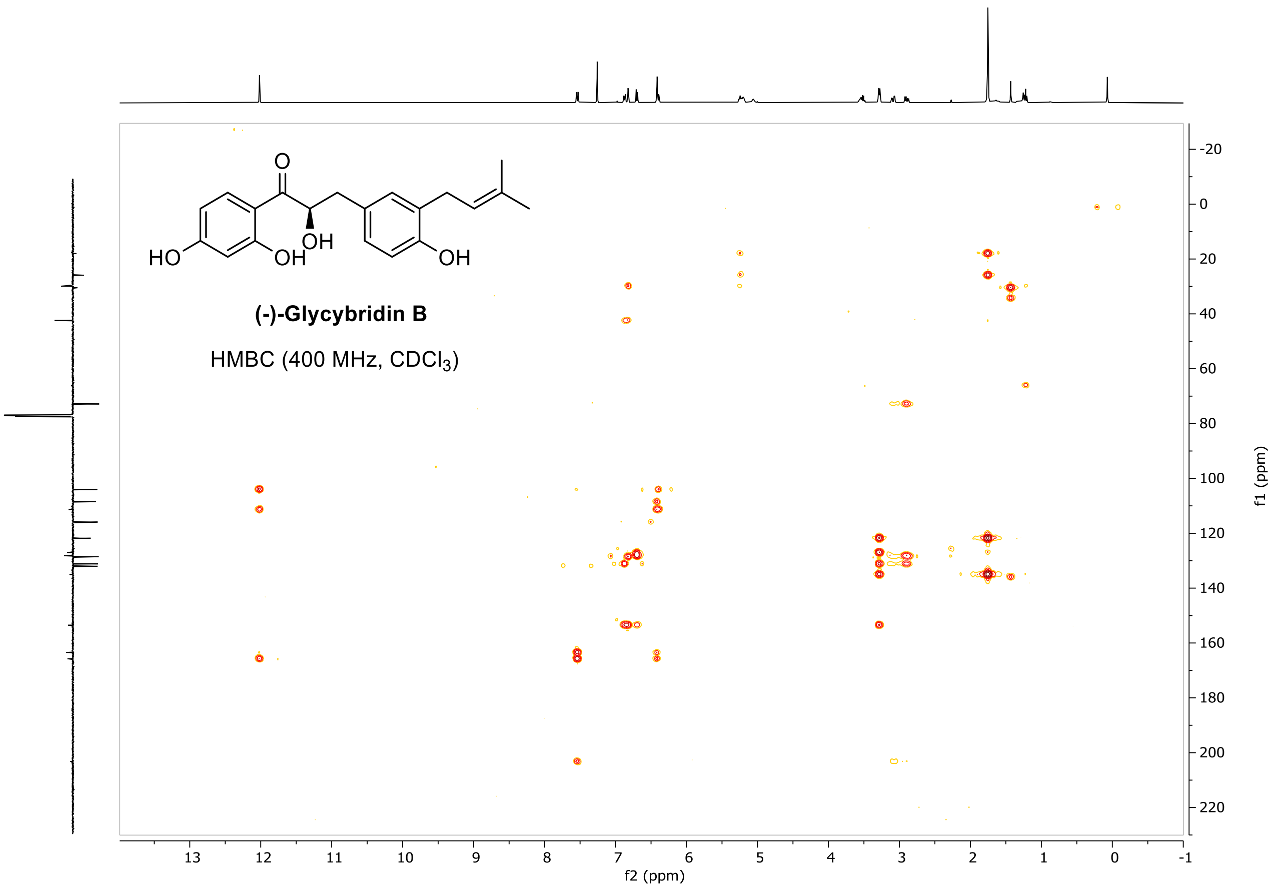

HO
OH
OH
O
OH
(-)-Glycybridin B
HMBC (400 MHz, CDCl3)
f1 (ppm)
f2 (ppm)

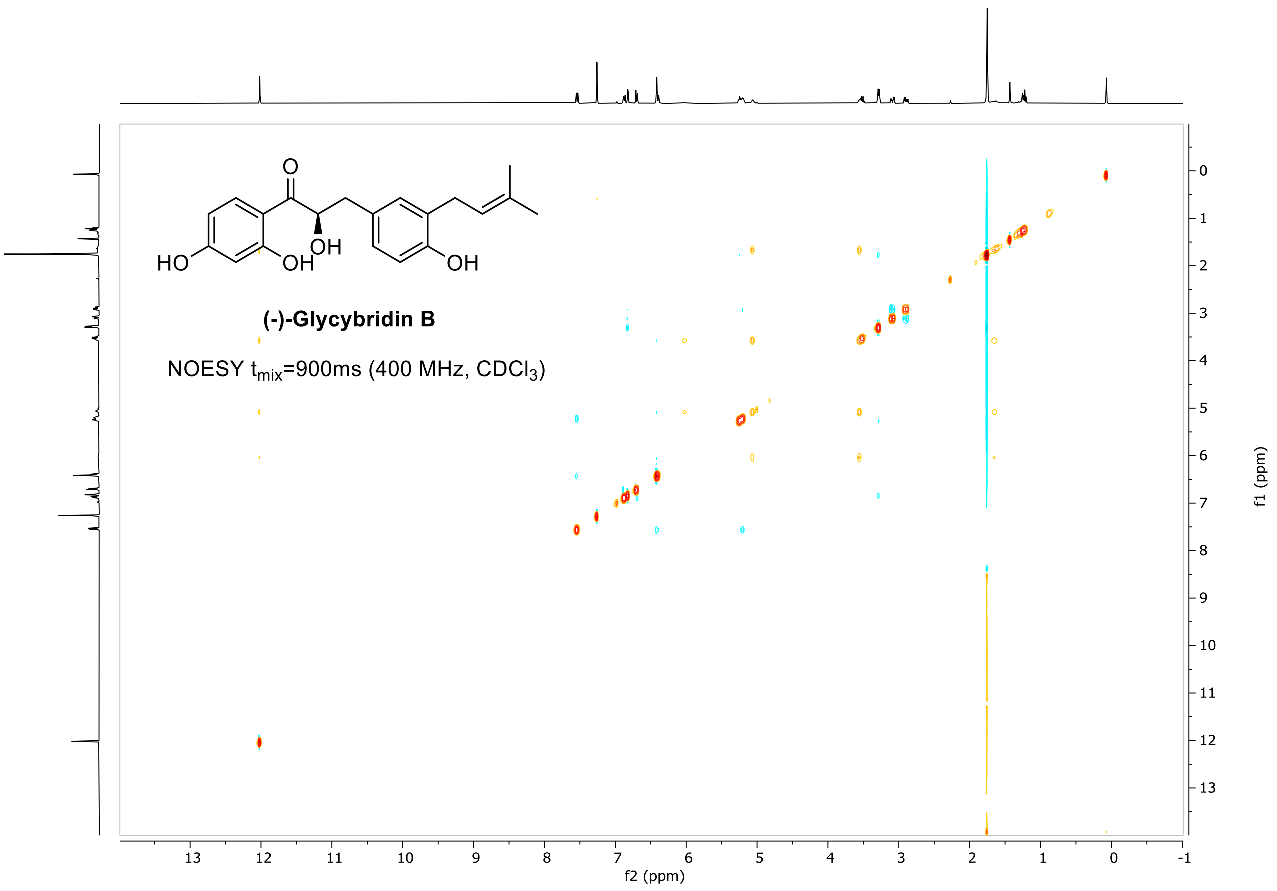

HO
OH
OH
O
OH
(-)-Glycybridin B
NOESY tmix=900ms (400 MHz, CDCl3)
f1 (ppm)
f2 (ppm)

HPLC chromatograms

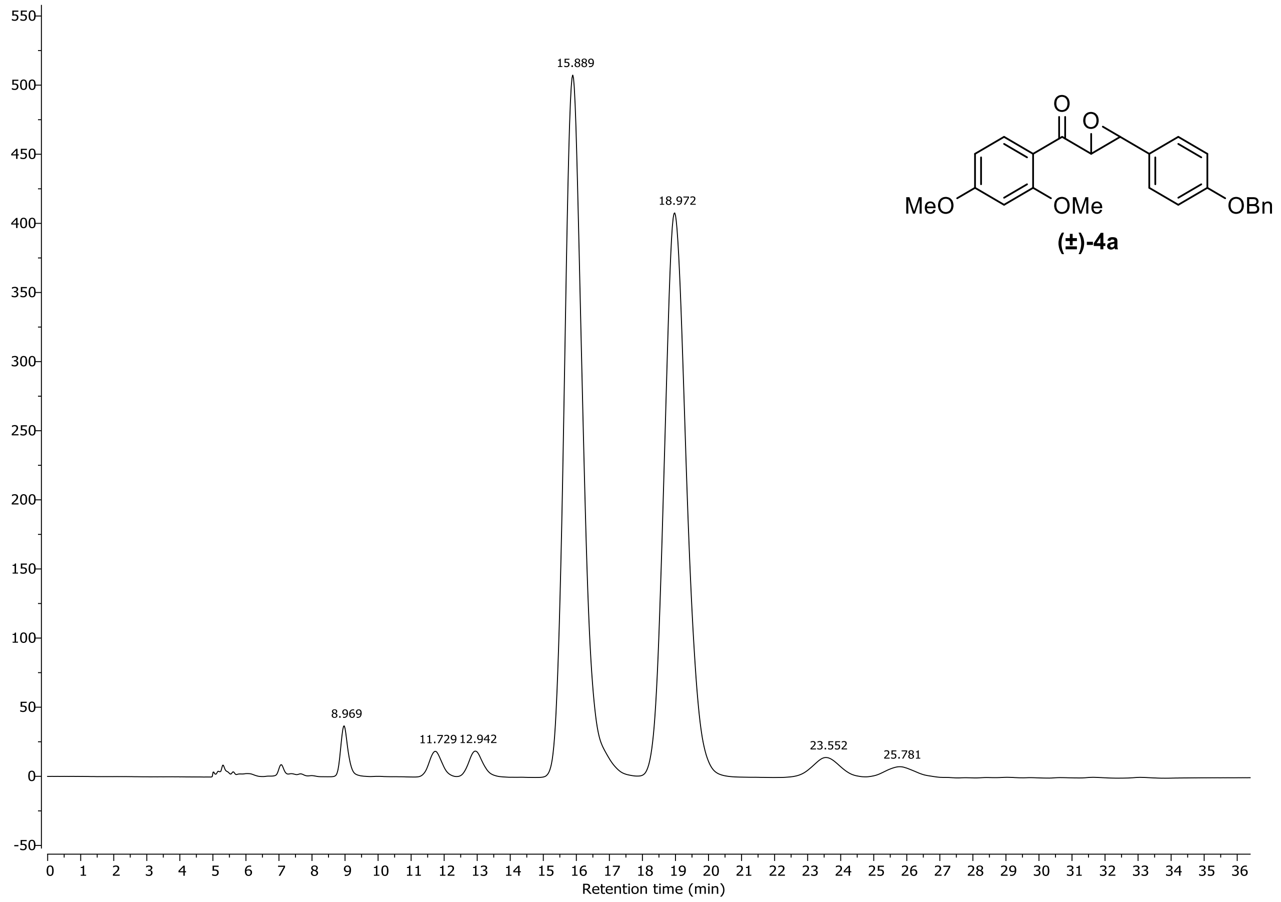

HPLC (Chiralpak AD, λ 220 nm, *n*-Hex/i-PrOH from 20:80 to 0:100, flow rate 0.8 mL/min): t_r(2S,3R) = 15.9 min, t_r(2R,3S) = 19.0 min.

Product	RT (min)	Area	Area %
**(+)-12a**	15.889	97617	48.20
**(-)-12a**	18.972	90902	44.89

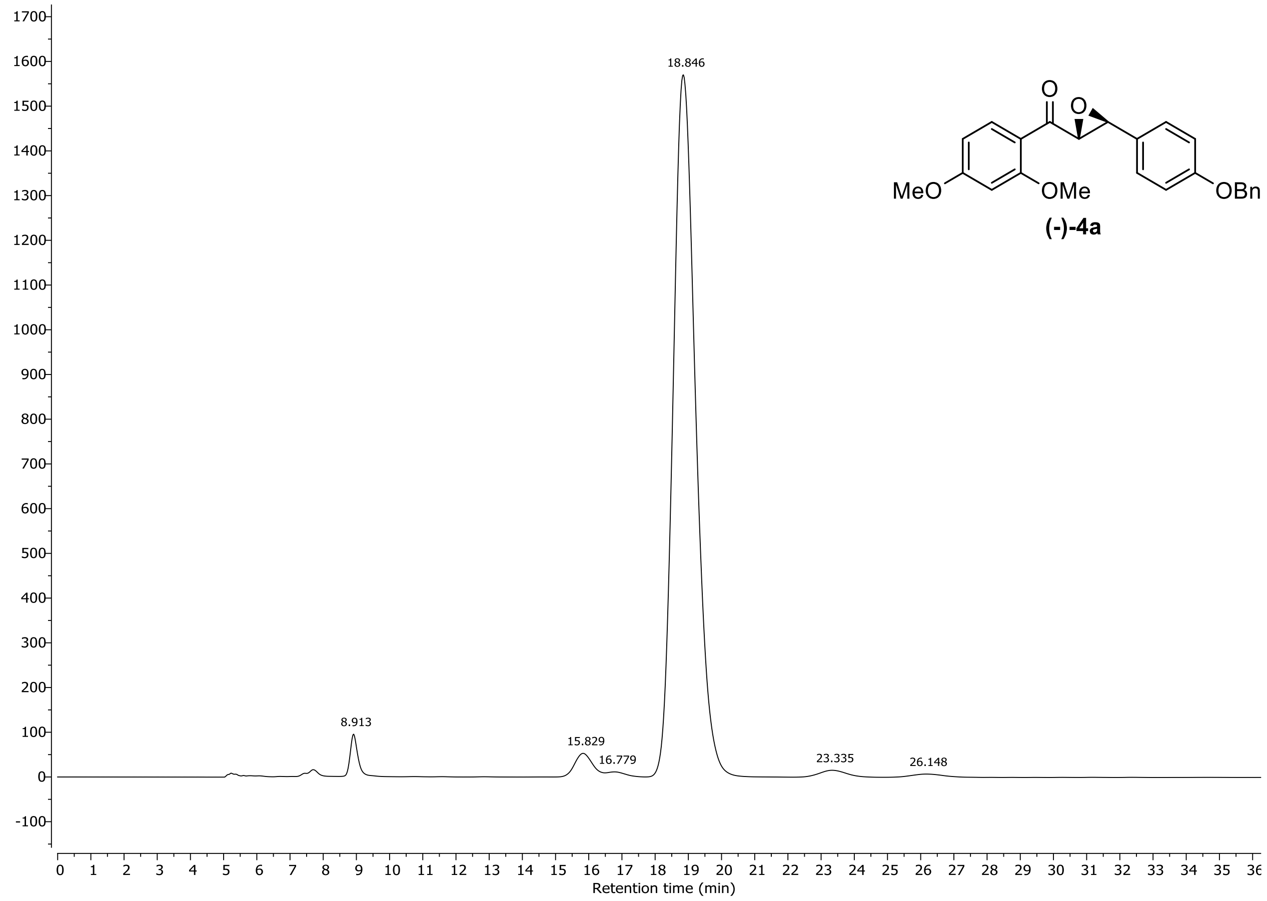

HPLC (Chiralpak AD, *n*-Hex/i-PrOH from 20:80 to 0:100, flow rate 0.8 mL/min): t_r(2S,3R) = 15.8 min, t_r(2R,3S) = 18.8 min.

Product	RT (min)	Area	Area %
**(+)-12a**	15.829	9937	2.59
**(-)-12a**	18.846	358319	93.36

ee%=95%

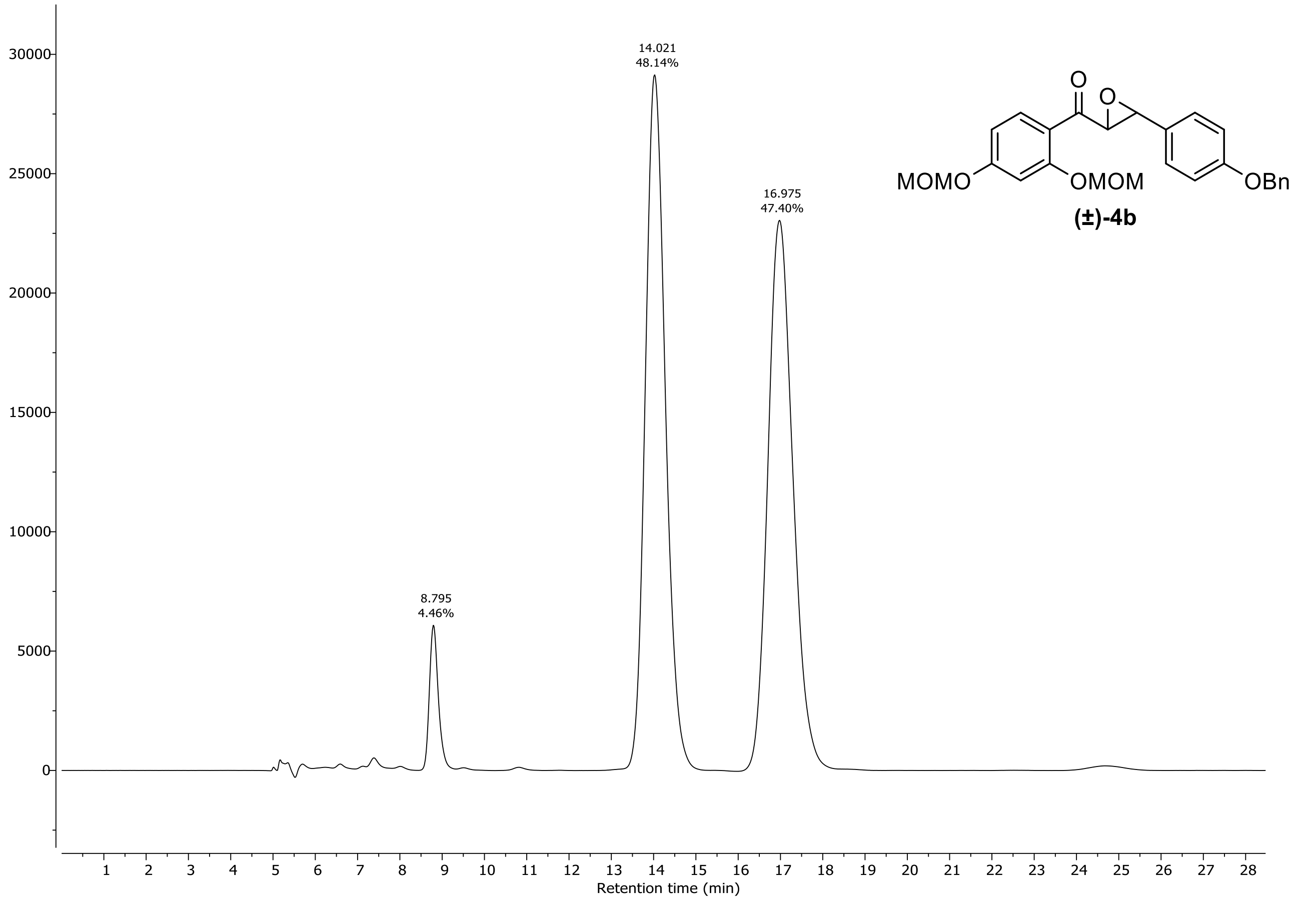

HPLC (Chiralpak AD, λ 220 nm, *n*-Hex/i-PrOH from 20:80 to 0:100, flow rate 0.8 mL/min): t_r(2S,3R) = 14.0 min, t_r(2R,3S) = 17.0 min.

Product	RT (min)	Area	Area %
**(+)-12b**	14.021	4619273	48.14
**(-)-12b**	16.975	4548229	47.40

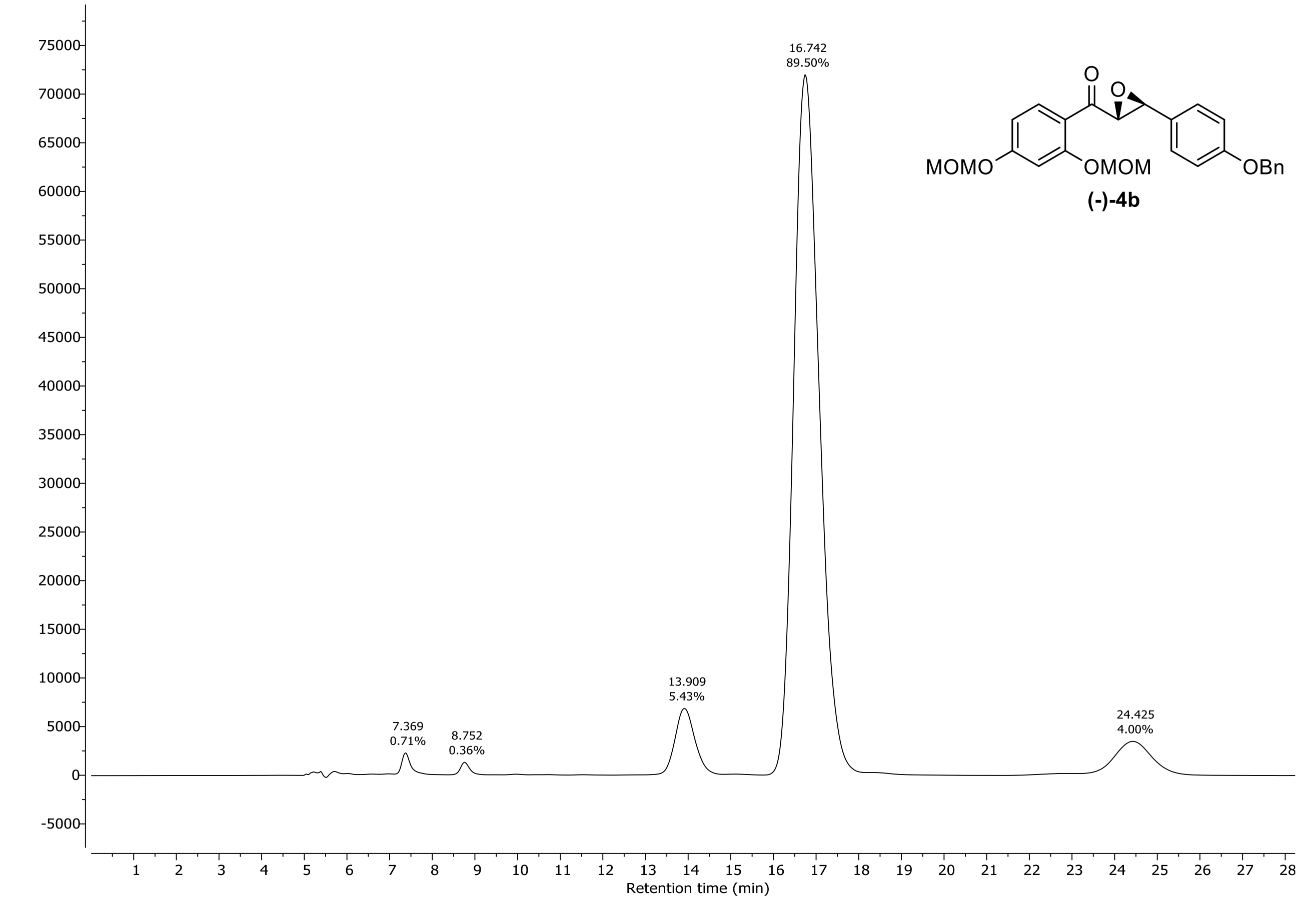

HPLC (Chiralpak AD, λ 220 nm, hexane/i-PrOH from 20:80 to 0:100, flow rate 0.8 mL/min): t_r(2S,3R) = 13.9 min, t_r(2R,3S) = 16.7 min.

Product	RT (min)	Area	Area %
**(+)-12b**	13.909	906504	5.43
**(-)-12b**	16.742	14939699	89.50

ee%=89%

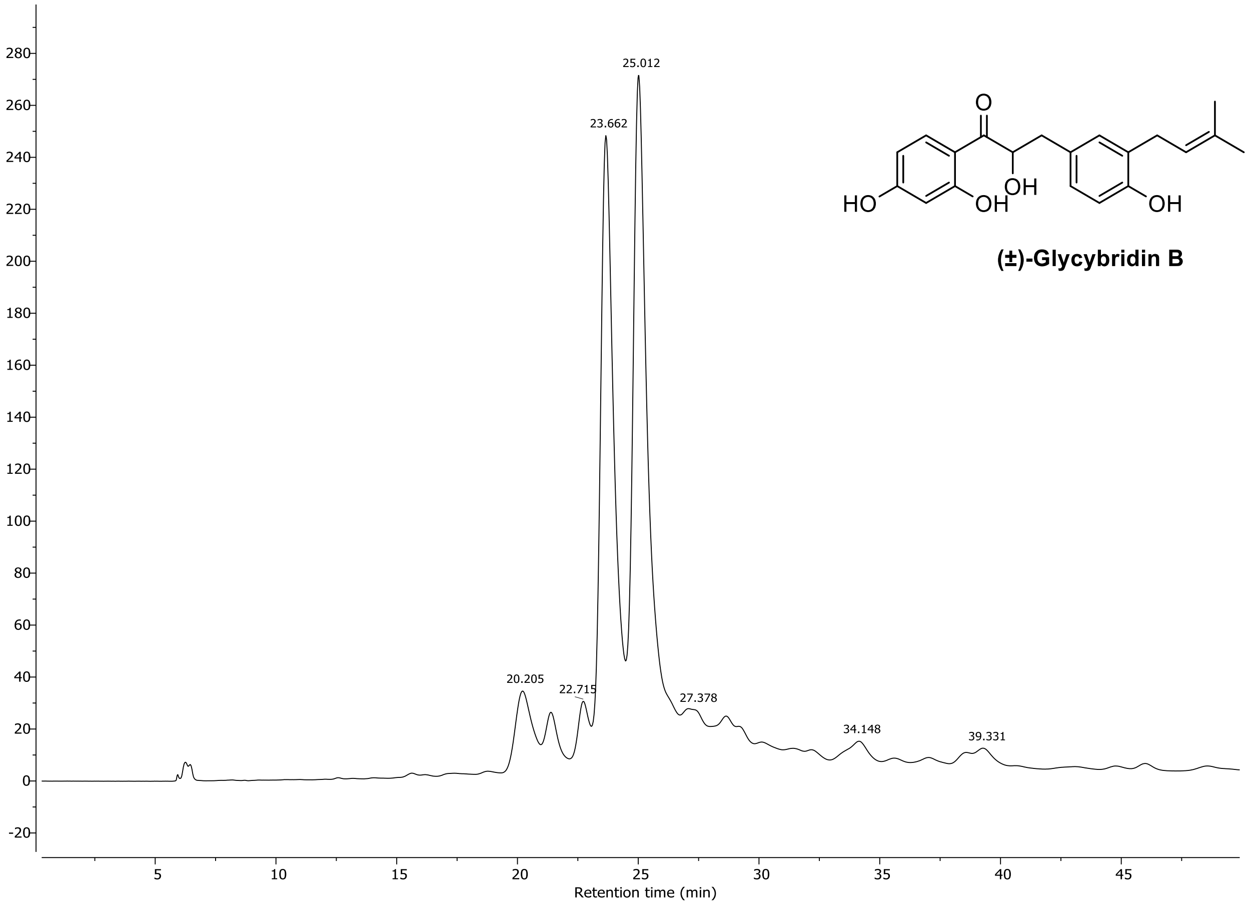

HPLC (Chiralpak AD, λ 280 nm, *n*-Hex/i-PrOH from 90:10 to 0:100, ramp 1%/min, flow rate 0.5 mL/min): t_r(2R) = 23.662 min, t_r(2S) = 25.012 min.

Product	RT (min)	Area	Area %
**(-)-Glycybridin B**	23.662	4264.836	40.59
**(+)-Glycybridin B**	25.012	46061.140	43.85

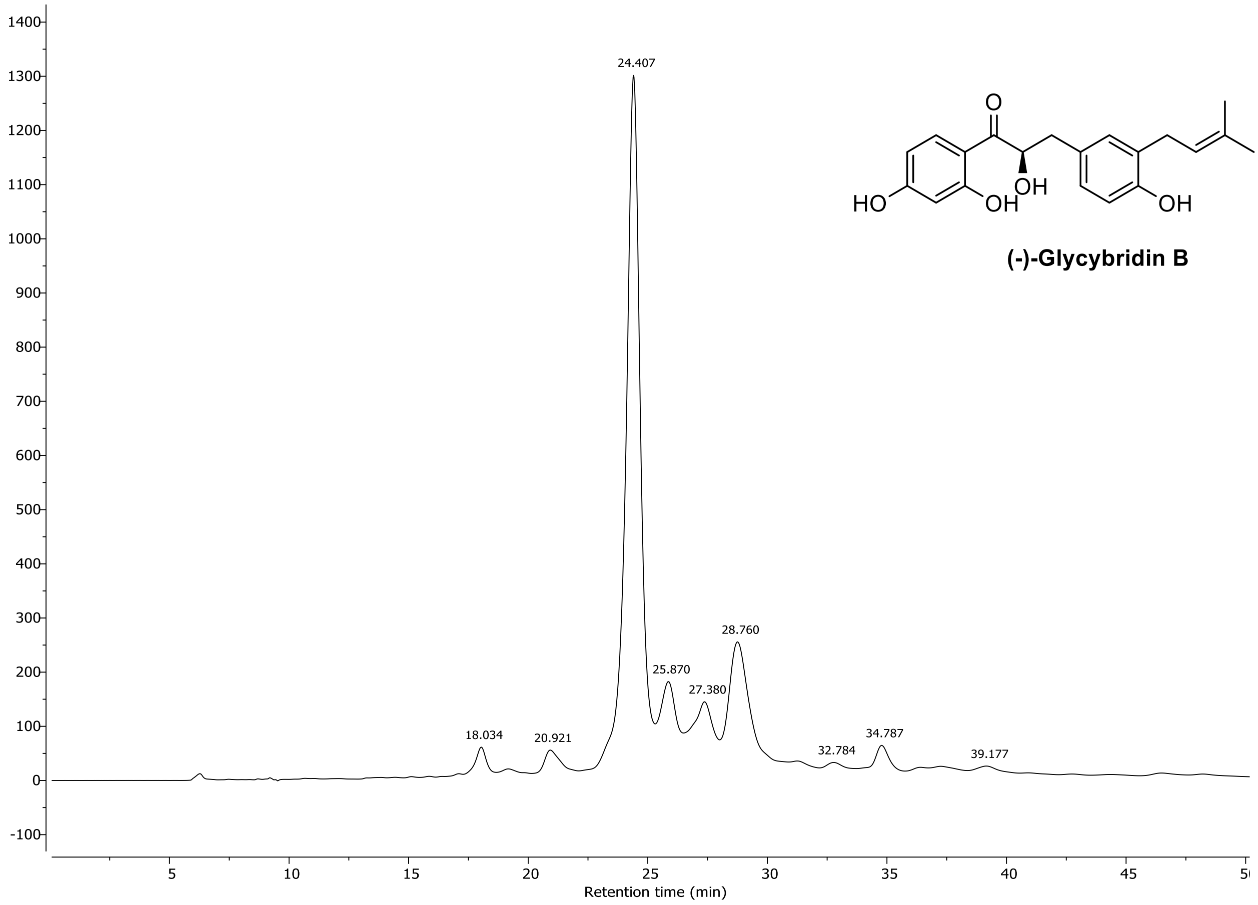

HPLC (Chiralpak AD, λ 280 nm, *n*-Hex/i-PrOH from 90:10 to 0:100, ramp 1%/min, flow rate 0.5 mL/min): t_r(2R) = 24.407 min, t_r(2S) = 25.870 min.

Product	RT (min)	Area	Area %
**(-)-Glycybridin B**	24.407	224306.892	68.74
**(+)-Glycybridin B**	25.870	13534.623	4.15

ee%=89%

Enantioenriched Glycybridin B mixed with around 0.1 eq of racemic compound in order to enhance the signal of the minor enantiomer

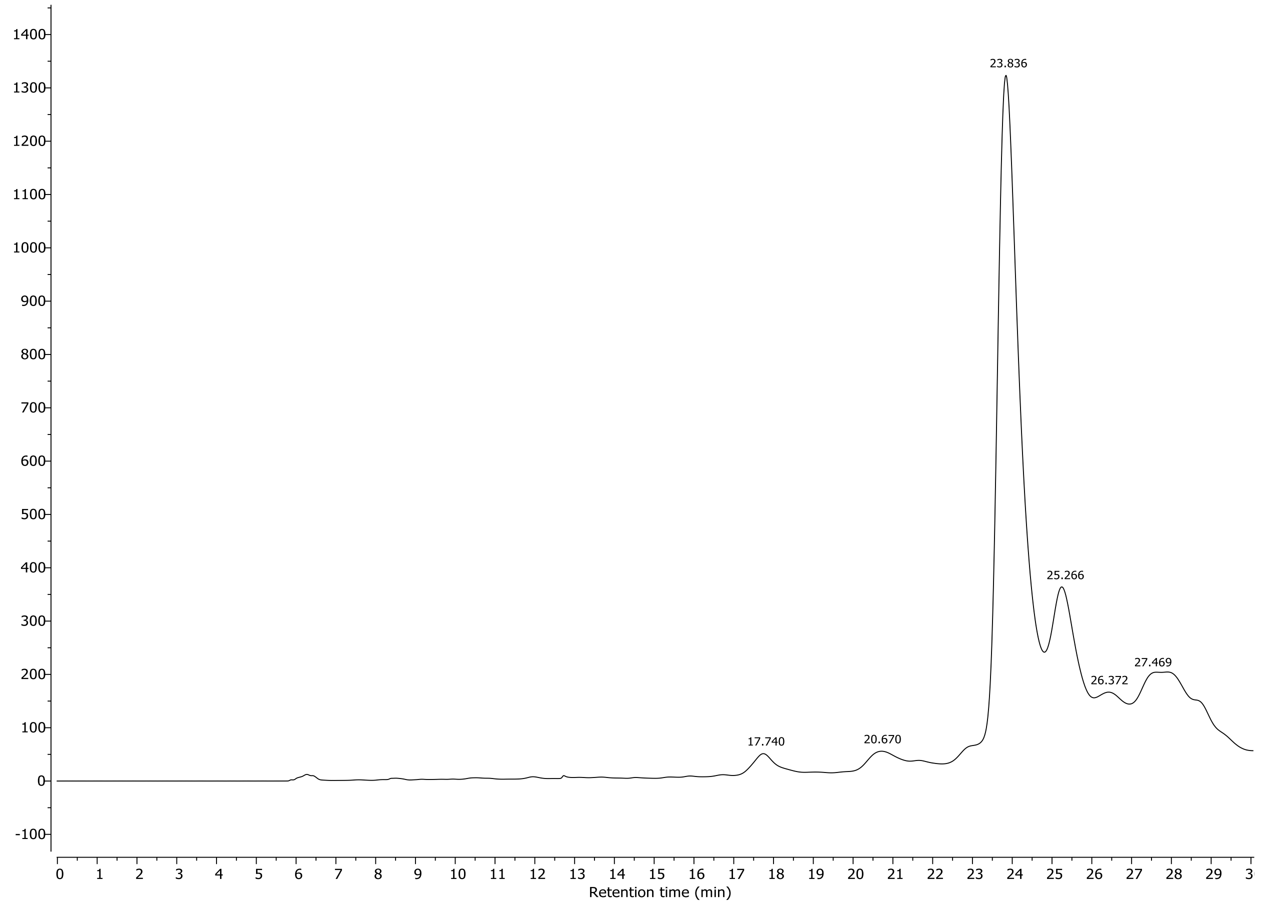

HPLC (Chiralpak AD, λ 280 nm, *n*-Hex/i-PrOH from 90:10 to 0:100, ramp 1%/min, flow rate 0.5 mL/min): t_r(2R) = 23.836 min, t_r(2S) = 25.266 min.

Product	RT (min)	Area	Area %
**(-)-Glycybridin B**	23.836	211061.203	81.89
**(+)-Glycybridin B**	25.266	24545.996	9.52

ee% of the sample=79%, in accordance with the spiking and confirming the chosen peak as the minor enantiomer peak.

Virtual Screening

Predicted binding mode of (*R*)-glycybridin B within the maytansine binding site of tubulin, as obtained from computational modeling studies. The supposed key molecular interactions are indicated.

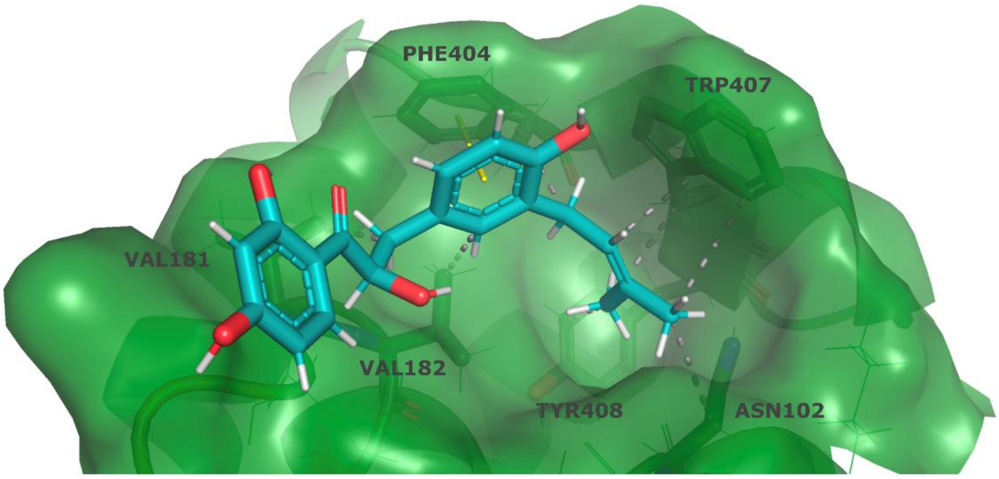

Supplement: Supplementary file 1 — Supplementary Information [file CHEM-31-e02228-s001.pdf]
